# Earthworms Enhance Global Soil Carbon Storage Through Microbial–Mineral Stabilization

**DOI:** 10.1111/gcb.70815

**Published:** 2026-03-19

**Authors:** Yuanyuan Li, Jiahui Liao, Peter B. Reich, Yu Fang, Jiajie Cao, Juanping Ni, Tingting Ren, Guobing Wang, Xiaoming Zou, Honghua Ruan, Han Y. H. Chen

**Affiliations:** ^1^ Joint Center for Sustainable Forestry in Southern China Nanjing Forestry University Nanjing People's Republic of China; ^2^ School of Food Science Nanjing Xiaozhuang University Nanjing People's Republic of China; ^3^ School for Environment and Sustainability, Institute for Global Change Biology University of Michigan Ann Arbor Michigan USA; ^4^ Department of Forest Resources University of Minnesota St. Paul Minnesota USA; ^5^ Institute of Agricultural Resources and Environment, Jiangsu Academy of Agricultural Sciences Nanjing China; ^6^ College of Grassland Science Inner Mongolia Agricultural University Hohhot China; ^7^ Faculty of Natural Resources Management Lakehead University Thunder Bay Ontario Canada

**Keywords:** biogeochemical cycling, earthworms, microbial efficiency, mineral‐associated organic carbon, soil aggregation, soil carbon stabilization

## Abstract

Earthworms play a dual role in the global carbon cycle: they accelerate organic matter decomposition yet are often associated with greater soil organic carbon (SOC) storage. However, uncertainty regarding the mechanisms and magnitudes through which earthworms concurrently influence SOC mineralization and stabilization has limited the integration of soil fauna into carbon models. Here, we synthesize 696 paired observations from 122 studies worldwide to resolve this uncertainty. On average, earthworms increase SOC by 5.4% (95% CI: 2.2%–9.1%), with effects strengthening over time under sustained plant‐derived carbon inputs. Earthworms enhance mineral‐associated organic carbon (MAOC) by 21.2%, while particulate organic carbon (POC) remains unchanged. These patterns suggest that earthworm activity promotes a transition from short‐term carbon mineralization to long‐term stabilization, likely mediated by the coupling of microbial processing and physical protection. Specifically, epigeic earthworms boost microbial biomass carbon, whereas endogeic species enhance macroaggregate formation, facilitating the incorporation of microbial necromass into MAOC. The magnitude and direction of these effects depend on sustained carbon inputs and earthworm functional type. Collectively, these results reconcile decades of conflicting evidence and provide the first quantitative global synthesis showing that earthworms increased soil carbon over time under sustained plant carbon inputs. This microbial–mineral formation pathway has direct implications for climate‐smart land management, soil biodiversity conservation, and the representation of earthworm bioturbation in global carbon models.

## Graphical abstract

Earthworms as conditional ecosystem engineers of the soil carbon cycle. The conceptual framework illustrates how earthworm effects on soil organic carbon (SOC) dynamics depend on plant‐derived carbon inputs and time. With sustained inputs, earthworm activity stimulates decomposition and nutrient release, boosting plant productivity. This synergy efficiently channels carbon into the stable mineral‐associated organic carbon (MAOC) pool, leading to net SOC accrual. Without inputs, their activity accelerates decomposition, causing net carbon loss.

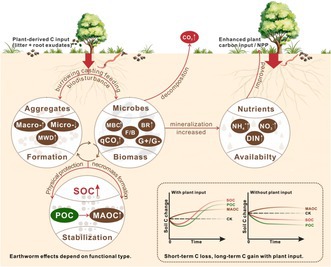

## 1 Introduction

Soils store more carbon than the atmosphere and all vegetation combined, making them both a critical buffer and a potential source in the global carbon cycle under accelerating climate change (Lal 2004; Delgado‐Baquerizo et al. 2017). Among the organisms regulating this vast carbon pool, soil fauna play key roles, with earthworms exerting particularly strong influences on soil processes (Bardgett and van der Putten 2014; Guidi et al. 2022; Angst et al. 2024). As ecosystem engineers, earthworms exert simultaneous effects on soil organic carbon (SOC): they accelerate organic matter decomposition and mineralization (Lubbers et al. 2013; Huang et al. 2020) while also enhancing the formation and stabilization of persistent SOC through microbial necromass production, organo‐mineral interactions, and physical aggregation (Bossuyt et al. 2005; Zhang et al. 2013; Angst et al. 2022; Calogiuri et al. 2025). This mechanistic perspective helps reconcile short‐term SOC losses (Lubbers et al. 2013) with evidence from longer‐term studies indicating potential net SOC gains over time (Zhang et al. 2013; van Groenigen et al. 2014). However, a unifying mechanistic framework is still needed to identify the ecological conditions under which earthworms transition from short‐term carbon mineralizers to longer‐term promoters of carbon retention.

**FIGURE 1:**
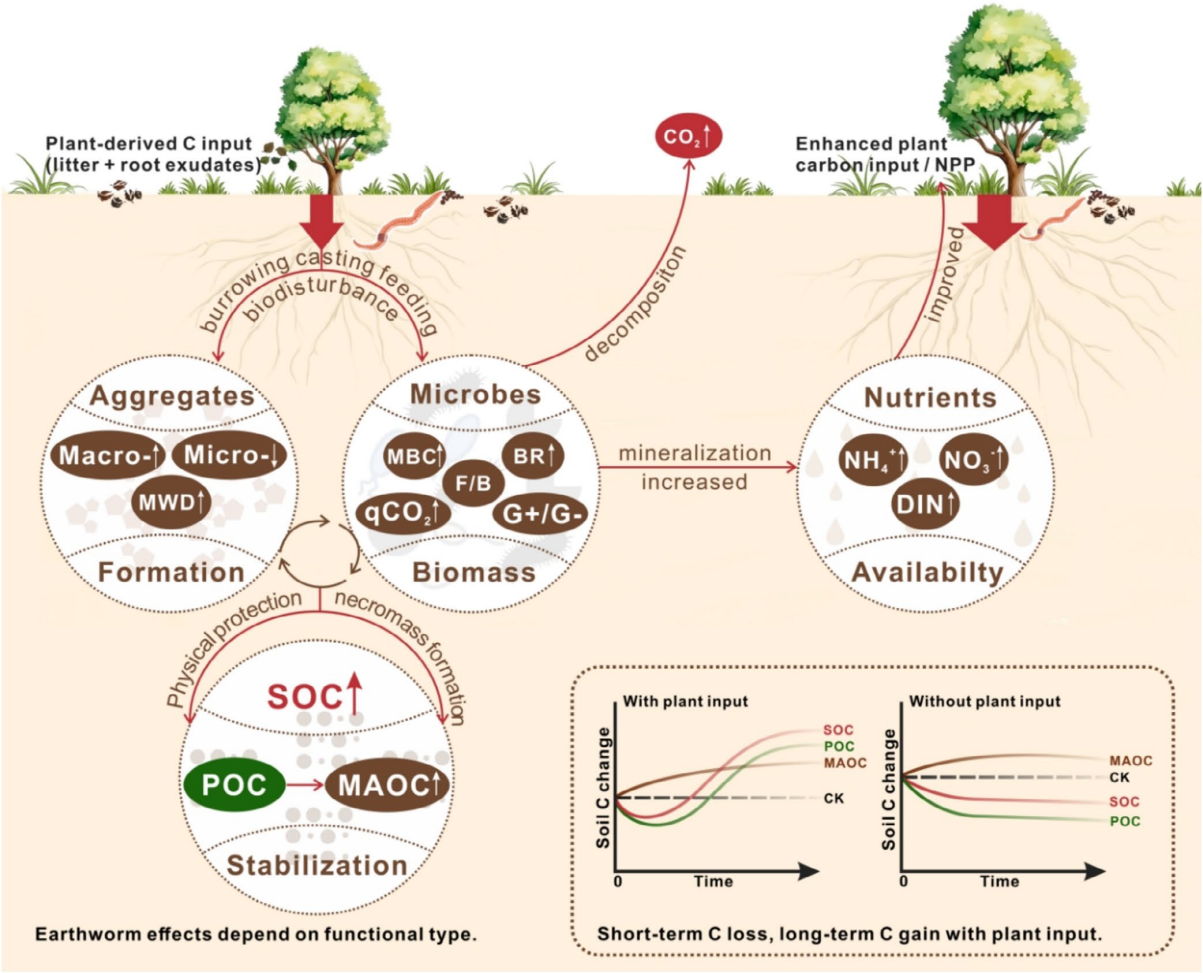
Earthworms as conditional ecosystem engineers of the soil carbon cycle. The conceptual framework illustrates how earthworm effects on soil organic carbon (SOC) dynamics depend on plant‐derived carbon inputs and time. With sustained plant inputs, earthworm activity (bioturbation, casting, feeding) stimulates microbial basal respiration and nutrient mineralization, which enhances plant productivity and increases POC inputs. This fuels a synergistic pathway where microbial processing transforms labile carbon into necromass, while earthworms simultaneously engineer stable macro‐aggregates and increase MWD. This co‐action efficiently channels carbon into the persistent MAOC pool, leading to net SOC accrual. Without plant inputs, earthworms enhance decomposition and respiration without replenishment, leading to net SOC loss. Arrows (↑ increase, ↓ decrease) indicate changes relative to an earthworm‐free control (CK). BR, microbial basal respiration; CK, control without earthworms; DIN, dissolved inorganic nitrogen; F/B, the fungal‐to‐bacterial ratio; G+/G–, Gram+: Gram– bacteria ratio; Macro‐, macro‐aggregates; MAOC, mineral‐associated organic carbon; MBC, microbial biomass carbon; Micro‐, micro‐aggregates; MWD, mean weight diameter; NH_4_^+^, ammonium nitrogen; NO_3_^−^, nitrate nitrogen; POC, particulate organic carbon; qCO_2_, microbial metabolic quotient.

We posit that resolving this pattern requires moving beyond decomposition rates alone to consider the interplay among carbon inputs, microbial processing, and physical stabilization (Figure 1). In the absence of plant‐derived substrates, earthworms enhance microbial activity without replenishing organic matter, thereby promoting SOC losses (Lubbers et al. 2013, 2017). In contrast, under continuous litter or root inputs, earthworms stimulate nutrient mineralization, increase particulate organic carbon (POC) inputs, and facilitate the subsequent stabilization of this carbon as mineral‐associated organic carbon (MAOC) through soil aggregation and organo‐mineral interactions (Bossuyt et al. 2005, 2006; Knowles et al. 2016; Frouz and Cathaml 2023). Earthworms also restructure microbial communities, favoring fast‐growing r‐strategists and Gram‐negative taxa (Drake and Horn 2007; Tao et al. 2009), while their casts and mucus redirect carbon fluxes from respiration toward microbial biomass production (Postma‐Blaauw et al. 2006; Wang et al. 2024). Over time, these coupled processes transform rapid carbon turnover into persistent SOC storage.

Yet, earthworm effects on SOC are highly context‐dependent, varying with functional type (epigeic, endogeic, anecic), community composition, and population density (Angst et al. 2017; Huang et al. 2020). For example, anecic species incorporate litter into macro‐aggregates, increasing early‐stage mineralization (Sheehy et al. 2019; Mao et al. 2024), whereas endogeic species preferentially embed microbial residues within stable micro‐aggregates, promoting longer‐term carbon stabilization (Angst et al. 2019; Mao et al. 2024). At low population densities, patchy bioturbation may destabilize soil structure and exacerbate SOC loss (Lubbers et al. 2013; Angst et al. 2024), while at moderate densities, communities comprising diverse functional types act synergistically to enhance MAOC formation and microbial necromass retention (Bossuyt et al. 2006; Huang et al. 2020; Ross et al. 2024). Despite these mechanistic insights, empirical inconsistencies persist due to variation in climate, ecosystem type, experimental setting, and earthworm origin (native vs. invasive) (Callaham et al. 2001; Zhang et al. 2013; Huang et al. 2015; Phillips et al. 2019; Edwards and Arancon 2022).

To synthesize these diverse findings and identify the dominant mechanisms, we conducted the most comprehensive global meta‐analysis of earthworm effects on SOC, compiling 696 paired observations from 122 peer‐reviewed studies across forests, grasslands, and cropland ecosystems. Each observation consisted of paired plots with and without earthworms that were otherwise comparable in soil type, vegetation, climate, and land‐use history, ensuring that SOC differences could be attributed to earthworm effects. Earthworms were categorized by functional type following established ecological classifications (anecic, epigeic, endogeic) (Lavelle 1988; Capowiez et al. 2024). We tested three hypotheses: (i) sustained plant‐derived carbon inputs enable earthworms to concurrently enhance microbial respiration and SOC persistence, primarily through increased MAOC and soil aggregation; (ii) these stabilization outcomes are governed by functional type, such that endogeic earthworms predominantly promote MAOC formation and aggregate stability, whereas epigeic earthworms exert stronger effects on initial decomposition pathways; and (iii) the magnitude of earthworm effects on SOC is systematically moderated by factors including earthworm origin, ecosystem type, and climate, with stronger net stabilization expected under higher carbon inputs and environmental conditions favorable for microbial–mineral interactions. By integrating functional type, ecosystem context, and temporal dynamics, our synthesis provides a process‐based framework for evaluating their contribution to long‐term soil carbon persistence, with direct implications for climate‐relevant management and carbon accounting.

## 2 Materials and Methods

### 2.1 Data Collection

We conducted a systematic literature search in Web of Science and Google Scholar using the keywords: “(earthworm OR earthworms) AND (soil organic carbon OR SOC OR particulate organic carbon OR POC OR mineral‐associated organic carbon OR MAOC OR microbial biomass OR fungal biomass OR bacterial biomass OR microbial respiration OR soil respiration OR basal respiration OR aggregate OR micro‐aggregate OR macro‐aggregate)”. Data on soil organic carbon, microbial communities, and soil aggregates were extracted directly from text, tables, and appendices, or digitized from figures using WebPlotDigitizer (https://automeris.io/WebPlotDigitizer/).

Studies were included if they met the following criteria: (1) reported SOC after a clearly defined experimental duration; (2) included both earthworm and control treatments under comparable abiotic and biotic conditions in laboratory or field settings; (3) involved direct earthworm inoculation into bulk soil (excluding studies focused solely on casts or burrow walls); (4) reported at least one of the following: microbial biomass carbon (MBC; mg C kg^−1^ soil; chloroform fumigation–extraction), basal respiration (BR; μg CO_2_—C g^−1^ soil day^−1^; soil incubation), microbial metabolic quotient (qCO₂ defined as CO₂ production per unit of microbial biomass carbon; mg CO_2_—C g^−1^ MBC h^−1^; calculated as BR/MBC), or soil aggregate mass fractions (g kg^−1^ soil; wet‐sieving: micro‐aggregates < 0.25 mm, macro‐aggregates > 0.25 mm, small macro‐aggregates 0.25–2 mm, large macro‐aggregates > 2 mm) or mean weight diameter (MWD; mm; dry‐sieving); (5) duplicated observations across publications were included only once; (6) studies using artificial soil mixes were excluded. Because 94% of incubation studies used soils from the top 20 cm, the soil depth exhibited insufficient variation to explain the between‐study differences meaningfully and was therefore excluded as a predictor.

Additional data were extracted for fungal biomass, bacterial biomass, fungal‐to‐bacterial (F:B) ratio, Gram‐positive‐to‐Gram‐negative (G^+^:G^−^) ratio, dissolved inorganic nitrogen (DIN), dissolved organic carbon (DOC), ammonium (NH_4_^+^—N), nitrate (NO_3_^−^—N), and soil pH. Climate variables—including mean annual temperature (MAT, °C) and mean annual precipitation (MAP, mm year^−1^)—were obtained from source publications or, when unavailable, from the WorldClim database (v.2.1) using site coordinates.

The final dataset comprised 696 paired observations from 122 peer‐reviewed studies (Figures S1 and S2; Table S1), with 25% conducted in the field and 75% as laboratory incubations. Although new definitions of earthworm functional types are available (Capowiez et al. 2024), we used the old definition to categorize earthworms into three functional types (epigeic, endogeic, anecic) based on feeding, burrowing, and casting traits (Lavelle 1988) because of the availability of data in old literature. Missing qCO_2_ values were imputed as BR/MBC where both were reported (Liu et al. 2023).

It should be noted that the earthworm functional classification applied here follows the widely used behavioral framework proposed by Lavelle (1988), rather than the more recent process‐based classification by Capowiez et al. (2024). Although species were classified as accurately as possible based on published descriptions, minor inconsistencies in species classification and functional assignment among studies may introduce uncertainty. Accordingly, our conclusions reflect response patterns defined within this established behavioral taxonomy.

### 2.2 Meta‐Analysis

Earthworm effects on soil organic carbon (SOC), microbial communities, and soil physicochemical properties were quantified using the natural log response ratio (lnRR) (Hedges et al. 1999).

(1) $$\text{lnRR}=\ln\left(\frac{X_{t}}{X_{c}}\right)$$

where $X_{t}$ and $X_{c}$ represent the mean values of the selected variable in treatments with and without earthworms, respectively.

For studies reporting measures of variability, sampling variance (*V*) was calculated following (Hedges et al. 1999):

(2) $$V=\frac{SD_{t}^{2}}{n_{t}X_{t}^{2}}+\frac{SD_{c}^{2}}{n_{c}X_{c}^{2}}$$

where $SD_{t}$ and $SD_{c}$ are the standard deviations of treatment and control groups, and $n_{t}$ and $n_{c}$ are corresponding sample sizes.

Because variability measures were not consistently reported across studies, we applied the All‐cases Method (Nakagawa, Noble, et al. 2023), which imputes missing standard deviations using maximum likelihood estimation based on weighted average coefficients of variation. This approach enables full data utilization while maintaining statistical validity.

To validate this procedure, we used SOC as a test case (89% of SOC observations reported variance). A sensitivity analysis comparing the Complete SD subset and the full dataset with imputed variances yielded statistically indistinguishable pooled effect sizes (*Z* = 0.433, *p* = 0.665; Figure S3a). Heterogeneity (*I* ^2^) was lower in the full dataset (75.1%) than in the Complete SD subset (96.3%; Figure S3b), indicating improved representativeness. Based on this validation, the All‐cases Method was applied uniformly to all response variables.

We fitted multilevel random‐effects models using restricted maximum likelihood (REML) in the rma.mv function of the *metafor* package (Viechtbauer 2010) in R v.4.3.2 (R Development Core Team 2023). The random structure was specified as:

~1 | studyID/observationID

to account for between‐study heterogeneity and within‐study non‐independence when multiple effect sizes were extracted from the same study (Nakagawa, Yang, et al. 2023). Effect sizes were weighted by the inverse of sampling variance (W = 1/V), as standard in meta‐analysis (Gurevitch et al. 2018). To ensure robust statistical inference, we applied Robust Variance Estimation (RVE) (Hedges et al. 2010), clustering by study ID to adjust standard errors, confidence intervals, and *p*‐values for correlated error structures.

Potential moderators were examined using mixed‐effects meta‐regression models:

(3) $$\text{lnRR}=\beta_{0}+\beta_{1}F+\pi_{\text{studyID}}+\pi_{\text{obsID}}+\varepsilon$$

where F represents moderator variables and π terms denote random effects. A full model including multiple predictors was also fitted:

(4) $$\begin{array}{c} \text{lnRR}=\beta_{0}+\beta_{1}P+\beta_{2}\ln\left(\mathrm{ED}\right)+\beta_{3}\ln\left(D\right)+\beta_{4}\text{FT}+\beta_{5}\text{IN}+\beta_{6}\mathrm{IT}\\ \;+\beta_{7}\mathrm{ET}+\pi_{\text{studyID}}+\pi_{\text{obsID}}+\varepsilon \end{array}$$

Predictors included carbon input source (P: live plant‐derived input, detrital organic matter input including litter or amendments, and no carbon input), experimental duration (ED), earthworm density (D), functional type (FT: endogeic, anecic, epigeic, or their mixtures), origin (IN: native or invasive species), study type (IT: field vs. laboratory incubation), and environmental variables (ET: ecosystem type, MAT, or MAP). Continuous predictors were log‐transformed where appropriate and standardized to improve model stability and comparability of coefficients.

Publication bias in SOC responses was assessed using Egger's regression test (Rothstein et al. 2005), implemented in *metafor* as a weighted regression of lnRR against its precision. The test was non‐significant (*p* > 0.05), and funnel plots showed no evidence of asymmetry (Figure S4).

Robustness of pooled effect sizes was further evaluated using leave‐one‐out sensitivity analyses (Viechtbauer 2010), sequentially removing each study and re‐estimating pooled effects under the same weighting and random‐effects structure (Figures S5 and S6). All statistical analyses were performed using R v.4.3.2 (R Development Core Team 2023).

## 3 Results

### 3.1 Average Earthworm Effects

**FIGURE 2:**
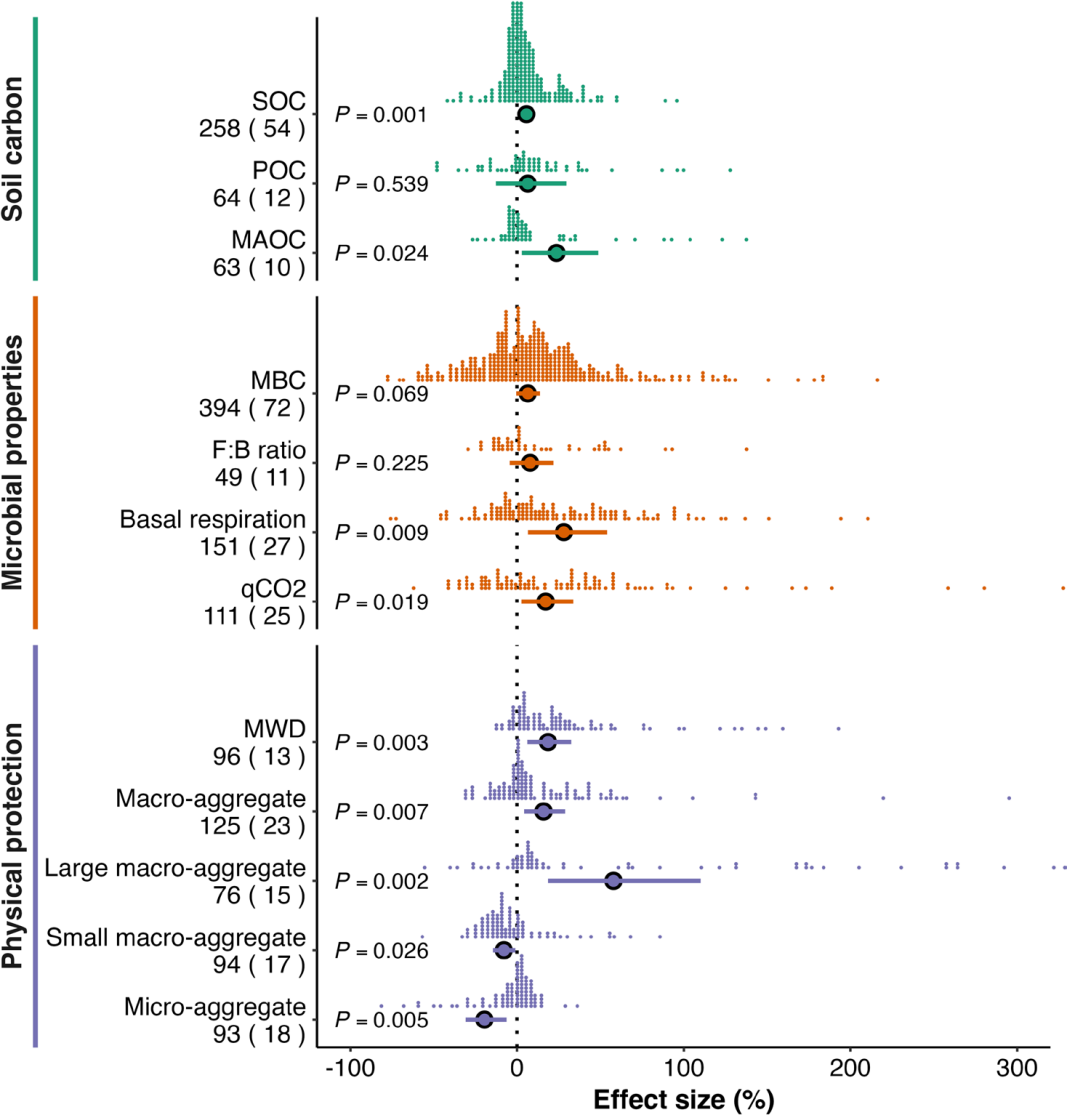
Effects of earthworms on soil organic carbon, microbial properties, and soil physical structure. Earthworm impacts are grouped into three categories: soil organic carbon (SOC), including SOC, POC, and MAOC (green); microbial properties (orange), including MBC, the fungal‐to‐bacterial (F:B) ratio, soil basal respiration (BR), and microbial etabolic quotient (qCO_2_); and soil physical properties (purple), including macro‐aggregates, small macro‐aggregates, large macro‐aggregates, micro‐aggregates, and MWD. Each dot represents a single observation, with horizontal position indicating effect size and vertical stacking indicating observation density. The number of observations is shown beside each attribute (unparenthesized), and the number of contributing studies is indicated in parentheses. Data are presented as mean ± 95% confidence intervals of percentage changes between earthworm and control treatments (*p* < 0.05).

Our global meta‐analysis shows that earthworms exert an overall positive effect on soil carbon storage across ecosystems. On average, earthworms increased MAOC by 21.2% (95% CI: 2.8%–48.6%, *p* = 0.024), whereas POC showed no significant change (+6.2%, 95% CI: −12.7%–29.6%, *p* = 0.539). Consequently, SOC stocks increased by 5.4% (95% CI: 2.2%–9.1%, *p* = 0.001; Figure 2). We also examined the effects of different moderators on SOC in a combined model to account for other factors, and the results remained qualitatively consistent with those from the individual analyses (Table S2). This net gain reflects the disproportionate contribution of the MAOC pool, which accounted for the overall increase despite the stability of POC.

Earthworm activity stimulated microbial metabolism, raising microbial basal respiration (BR) by 24.7% (95% CI: 6.5%–54.0%, *p* = 0.009) and the microbial metabolic quotient (qCO_2_) by 15.8% (95% CI: 2.6%–33.7%, *p* = 0.019). Microbial biomass carbon (MBC) showed a marginal increase of 6.2% (95% CI: −0.5%–13.8%, *p* = 0.069), whereas fungal and bacterial biomass, fungal‐to‐bacterial (F:B), and Gram‐positive‐to‐Gram‐negative ratios (G^+^:G^−^) remained unchanged.

In addition, earthworms reorganized soil physical structure. They enhanced mean aggregate diameter (MWD) by 17.0% (95% CI: 6.1%–32.5%, *p* = 0.003), and increased the proportion of macro‐aggregates and large macro‐aggregates (> 2 mm) by 14.7% (95% CI: 4.2%–28.8%, *p* = 0.007) and 45.6% (95% CI: 18.5%–110.1%, *p* = 0.002), respectively. In contrast, small macro‐aggregates and micro‐aggregates declined by 8.3% (95% CI: −14.4% to −9.7%, *p* = 0.026) and 21.8% (95% CI: −31.0% to −6.4%, *p* = 0.005).

### 3.2 Modulation by Carbon Input and Experimental Duration

**FIGURE 3:**
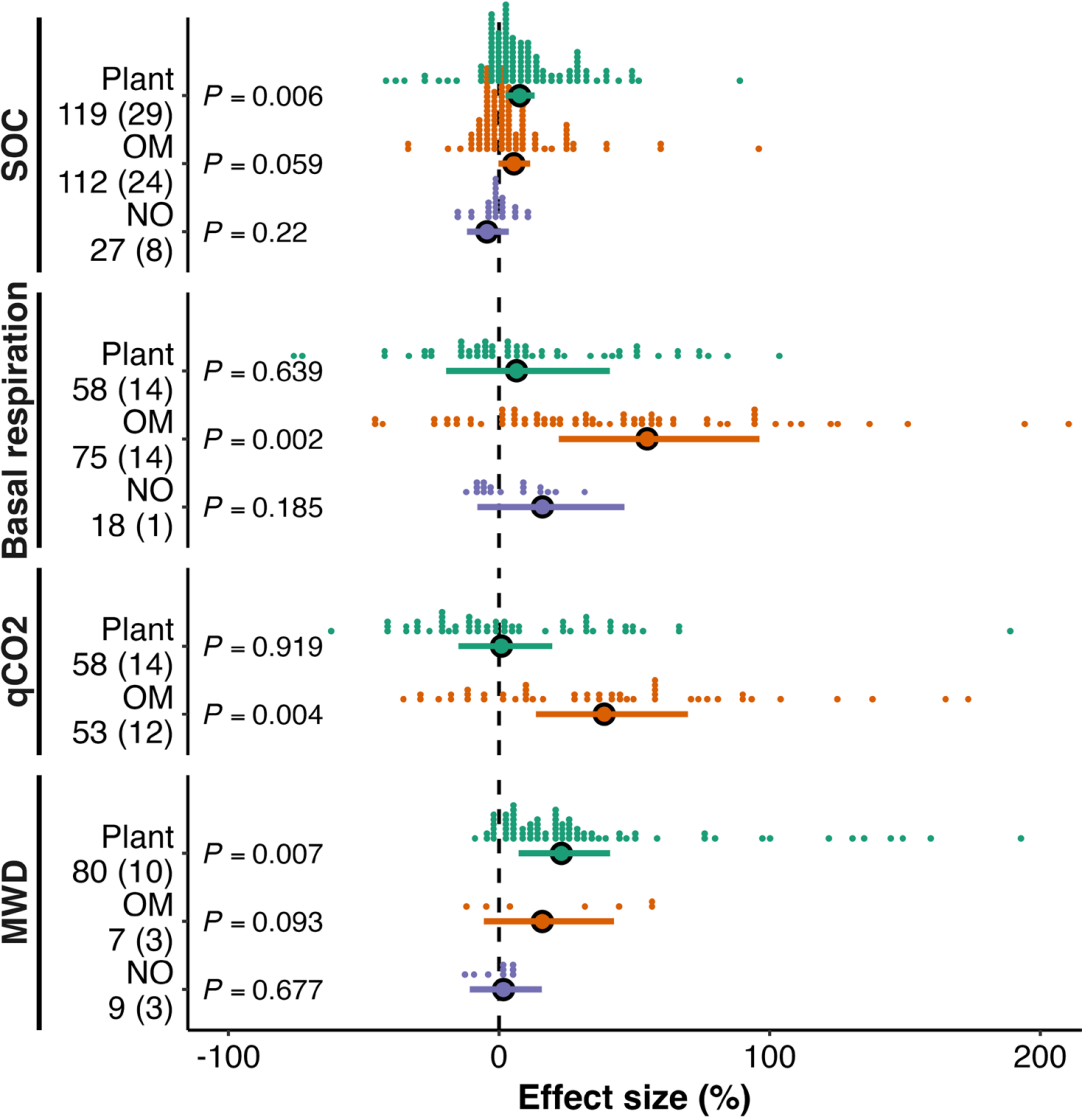
Earthworm effects on SOC and microbial processes depend on plant input type. Responses of soil organic carbon (SOC), soil basal respiration (BR), microbial metabolic quotient (qCO_2_), and mean weight diameter (MWD) are shown across different plant input types (Plant: live plant‐derived input, OM: detrital organic matter input (litter or amendments), and NO: no carbon input). Values represent the mean ± 95% confidence intervals of percentage changes between earthworm and control treatments. Each dot represents a single observation, with horizontal position indicating the effect size and vertical stacking reflecting observation density. The number of observations is indicated beside each attribute (without parentheses), and the number of contributing studies is shown in parentheses.

The effect of earthworms on SOC was contingent on the carbon input source. Earthworms increased SOC by 7.6% (95% CI: 2.3%–13.1%, *p* = 0.006) under continuous input (living plants) and by 5.4% (95% CI: −0.2%–11.4%, *p* = 0.059) under discrete input (detritus). In contrast, earthworms had a slight but non‐significant decrease in SOC without carbon input (−4.5%, 95% CI: −11.9%–3.5%, *p* = 0.22). The response of the microbial metabolic quotient and basal respiration increased under discrete input (detritus) (Figure 3). The mean weight diameter (MWD) of soil aggregates increased by 23.0% (95% CI: 7.3%–41.0%, *p* = 0.007) under continuous input (live plants).

**FIGURE 4:**
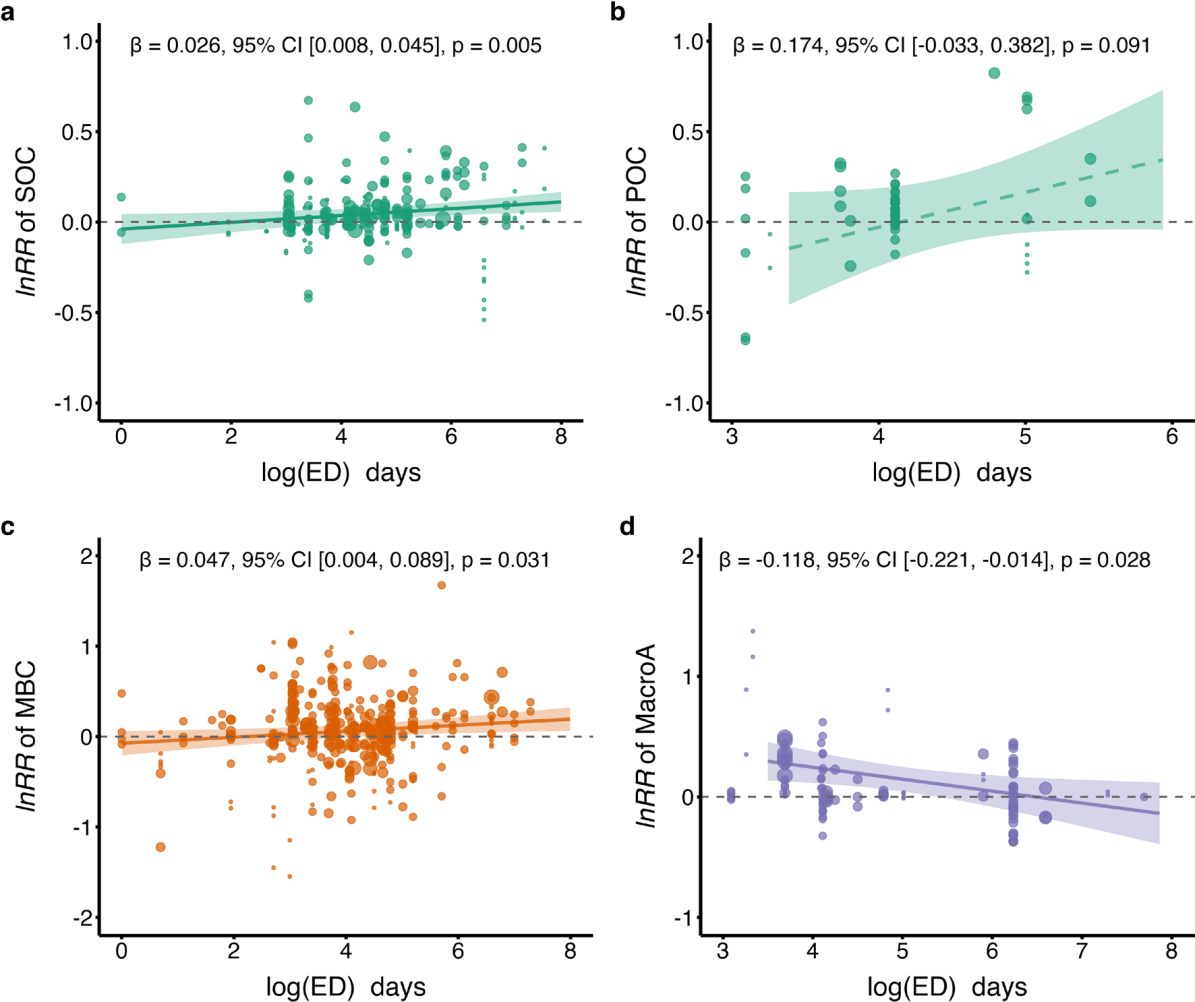
Bivariate relationships between experimental duration and key soil responses to earthworms. Partial dependence plots illustrate the relationships between experimental duration (ED, in days) and log response ratios (lnRR) of (a) soil organic carbon (SOC), (b) particulate organic carbon (POC), (c) microbial biomass carbon (MBC), and (d) macro‐aggregates under earthworm treatments, based on multivariate meta‐regression models. Here, experimental duration refers to the total period of earthworm exposure reported in each primary study, encompassing both incubation (cultivation) experiments and litter decomposition experiments. Each filled circle represents an observation, scaled by its inverse‐variance weight (W = 1/V) in the meta‐analysis. Solid or dashed lines indicate the predicted meta‐regression fit. The slope (*β*) with its 95% confidence interval (CI) and *p*‐value (*p*) for the experimental duration effect are annotated in each panel.

**FIGURE 5:**
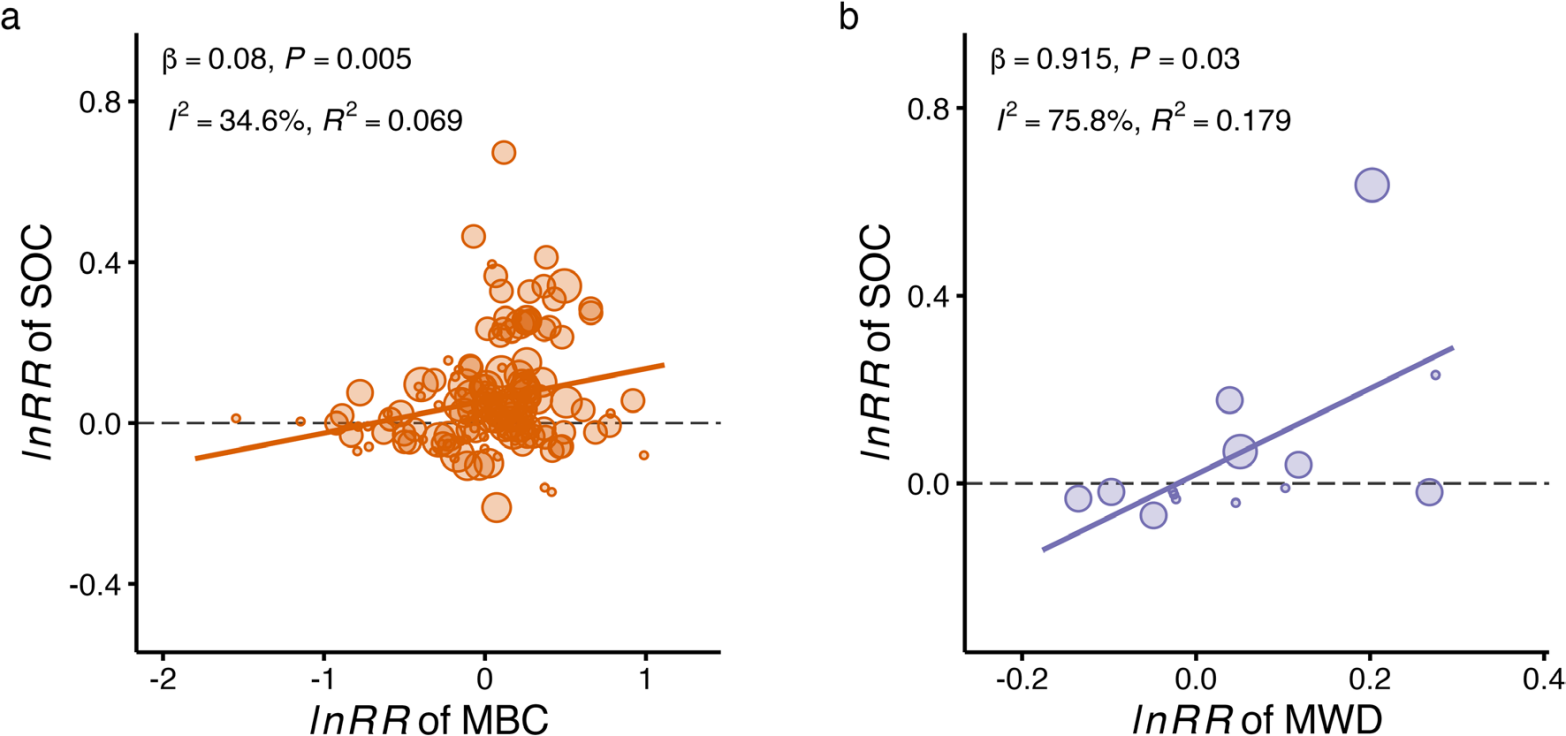
Bivariate relationships between soil organic carbon (SOC) and associated soil variables under earthworm treatments. Log response ratios (lnRR) of (a) microbial biomass carbon (MBC) and (b) mean weight diameter (MWD) as functions of the lnRR of SOC across all studies with earthworm inoculation. Each filled circle represents an observation, scaled by its inverse‐variance weight (W = 1/V) in the meta‐analysis. Solid lines indicate the predicted meta‐regression fit. The regression slope (*β*), its corresponding *p*‐value (*p*), the heterogeneity index (*I* ^2^), and the marginal coefficient of determination (*R* ^2^) are annotated in each panel.

Across the entire dataset, the magnitude of earthworm effects on SOC and microbial biomass carbon (MBC) increased linearly with the logarithm of experimental duration (Figure 4a,c), indicating a cumulative process rather than a transient response. This positive temporal trend was observed primarily in the presence of carbon inputs (Figure 3), where significant SOC increases occurred. Moreover, SOC responses to earthworms were positively correlated with changes in both MBC and mean weight diameter (MWD) (Figure 5), linking temporal amplification to microbial biomass expansion and aggregate stabilization.

### 3.3 Influence of Earthworms' Functional Type and Density

**FIGURE 6:**
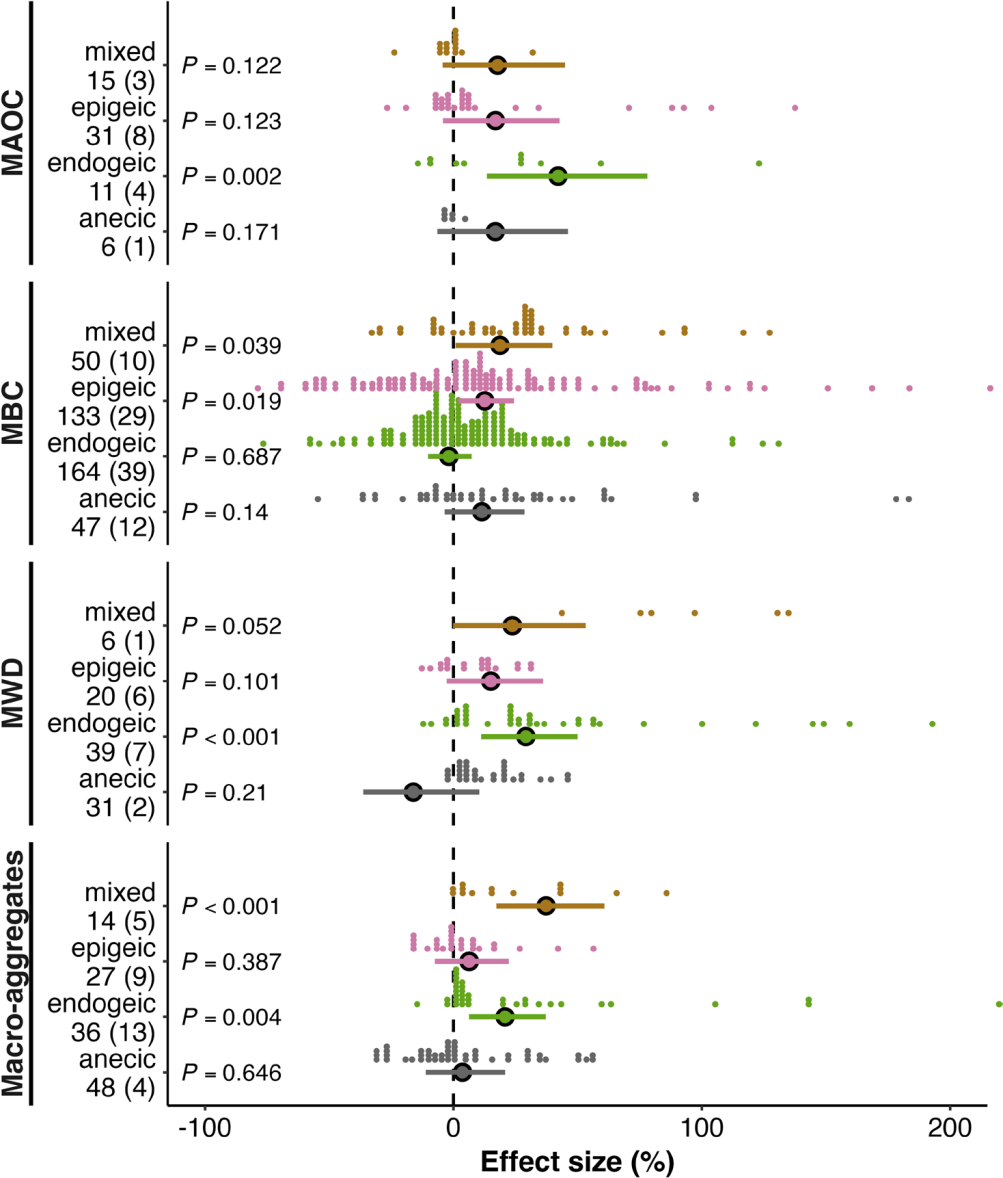
Effects of earthworm functional types on microbial biomass and soil structure. Responses of mineral‐associated organic carbon (MAOC), microbial biomass carbon (MBC), mean weight diameter (MWD), and macro‐aggregate fractions to different earthworm ecological functional types. Values represent the mean ± 95% confidence intervals of percent changes relative to control treatments. Each dot corresponds to an individual observation; horizontal placement reflects the effect size, and vertical stacking indicates observation density. The number of observations is listed beside each response variable (no parentheses), and the number of distinct studies contributing to each response is shown in parentheses.

The impact of earthworms on MBC and the soil aggregates varied significantly depending on earthworm functional type, whereas their impact on mineral‐associated organic carbon (MAOC) was consistent across earthworm functional type (Figure 6). Specifically, endogeic species significantly increased MAOC by 42.2% (95% CI: 13.5%–78.0%, *p* = 0.002). Epigeic species significantly increased MBC by 12.6% (95% CI: 1.9%–24.4%, *p* = 0.019), and mixed‐species assemblages increased MBC by 18.8% (95% CI: 0.9%–39.9%, *p* = 0.039). While endogeic and anecic species showed no significant effects. Both endogeic and mixed‐species assemblages significantly increased MWD by 29.2% (95% CI: 11.3%–50.0%, *p* < 0.001) and 23.7% (95% CI: −0.1%–53.3%, *p* = 0.052), respectively. Consistently, macro‐aggregate formation was promoted under endogeic species (+20.8%, 95% CI: 6.3%–37.3%, *p* = 0.004) and mixed assemblages (+37.3%, 95% CI: 17.3%–60.8%, *p* < 0.001).

## 4 Discussion

Our global meta‐analysis provides compelling evidence that earthworms, as ubiquitous ecosystem engineers, are significant drivers of soil carbon sequestration across terrestrial ecosystems. The net SOC increase, observed in 64% of all paired comparisons, demonstrates that earthworms generally act as carbon enhancers rather than sources. In the context of global change, where soils remain pivotal yet uncertain components of the carbon cycle, these findings reveal a biological mechanism capable of influencing both the direction and magnitude of land and atmosphere carbon feedbacks. By demonstrating that earthworms drive a net increase in SOC through microbial–mineral stabilization, our synthesis helps reconcile contrasting findings in previous studies and positions earthworms as key modulators of the global soil carbon sink.

### 4.1 A Synergistic Mechanism for SOC Stabilization

The net SOC increase reflects earthworms' simultaneous acceleration of carbon turnover and redirection of carbon into stabilized forms. Earthworms stimulate decomposition and nutrient mineralization (e.g., ammonium, *p* < 0.001; nitrate, *p* = 0.013; Figure S7), increasing short‐term respiration. However, they also promote microbial necromass production and enhance physical protection through aggregate formation and organo–mineral associations (Bossuyt et al. 2006; Angst et al. 2021). Consequently, the cumulative stabilization of processed carbon within mineral‐associated pools can exceed transient respiratory losses, resulting in a net SOC gain even under fixed carbon inputs.

Critically, earthworms primarily allocate newly incorporated carbon into the mineral‐associated organic carbon (MAOC) pool rather than into particulate organic carbon (POC). This occurs through bioturbation, mucus secretion, and casting, which promote the formation and persistence of macro‐aggregates (Angst et al. 2024). Although earthworm activity can transiently destabilize SOC by disrupting pre‐existing aggregates (Sánchez‐de León et al. 2014; Wu et al. 2017; Ma et al. 2022), the net effect is a sustained cycle of aggregate turnover that progressively reallocates carbon into mineral‐associated pools (Bailey et al. 2019; Mao et al. 2024). This mechanism is directly supported by our observed increase in macro‐aggregate fractions accompanied by a corresponding decline in micro‐aggregates (Figure 2).

Collectively, earthworms act as ecosystem engineers that enhance SOC through these coupled processes. This aligns with Olson's principle (Olson 1963), wherein SOC accrual co‐occurs with accelerated decomposition (*k*, which is proportional to the ratio of plant litter input to residual plant litter mass) when carbon inputs are sufficiently amplified. Within this framework, earthworms facilitate the transformation of labile carbon into stable MAOC, enabling a net increase in carbon storage despite heightened microbial respiration. Our results provide direct support: a 5.4% increase in SOC co‐occurred with a 24.7% rise in microbial respiration. This gain is ecologically meaningful against global SOC dynamics, where MAOC turnover is slow (mean ~ 129 years) and subsoil SOC persists for centuries (mean ~ 1015 years) (Luo et al. 2019; Zhou et al. 2024). Against this backdrop of generally slow turnover, a 5.4% accumulation underscores earthworms' ability to significantly perturb the soil carbon cycle within years to decades.

### 4.2 Regulation by Carbon Input and Experimental Duration

Our global meta‐analysis revealed that the net effects of earthworms on SOC were contingent on both the source of carbon input and the duration of the experiment. Positive SOC effects intensified over time, particularly under continuous plant inputs (Figure S8). This dependency was mechanistically linked to shifts in microbial metabolic efficiency. Under continuous inputs, the stability of microbial metabolic quotient (qCO_2_), coupled with a significant increase in microbial biomass carbon (MBC) (Figure 4c), suggests that microbial growth kept pace with respiratory carbon loss. This pattern likely reflects abundant carbon availability, under which microbial communities concurrently increase biomass production and respiration (Hu et al. 2025), thereby channeling a greater absolute flux of carbon into biomass. The significant positive correlation between SOC and MBC (Figure 5) indicates that microbial biomass expansion is associated with SOC accrual. We therefore propose that sustained carbon availability promotes microbial biomass accumulation, which may facilitate subsequent stabilization through necromass formation and mineral association (Liang et al. 2017; Hu et al. 2025), ultimately contributing to SOC accumulation (Tao et al. 2023; Wang et al. 2024).

Notably, earthworm effects on macroaggregates decreased with experimental duration (Figure 4d), suggesting accelerated aggregate turnover under prolonged bioturbation. Despite this decline in macroaggregate fraction, mean weight diameter (MWD) showed an increasing trend in the presence of plants (Figure 3). This apparent paradox likely arises from the formation of fewer but more stable aggregates, a process enhanced by microbial activity (Qiang et al. 2023). The positive correlation between MWD and SOC (Figure 5b) confirms that long‐term stabilization depends on the sustained formation of stable aggregates, co‐engineered by earthworms and microbial processes (Angst et al. 2024).

Our findings demonstrate that the net impact of earthworms on soil carbon depends both on carbon input availability and time. While the individual roles of substrate supply and experimental duration have often been examined separately, our synthesis integrates them into a predictive framework. Short‐term studies or those with limited carbon inputs (e.g., single litter additions) frequently emphasize the initial mineralization phase and report neutral or negative SOC responses (Lubbers et al. 2013; Huang et al. 2020). In contrast, under sustained inputs, the initial stimulation of decomposition and respiration is followed by a slower phase of net SOC accrual. This delayed stabilization likely reflects shifts in microbial community composition toward more fungal‐dominated assemblages (Figure S9), gradual incorporation of processed carbon into persistent organo–mineral associations (Bossuyt et al. 2005; Angst et al. 2022), and progressive reorganization of soil structure consistent with aggregate hierarchy theory (Six et al. 2002; Pulleman et al. 2005). Together, these processes enhance the physical and biochemical protection of carbon within stable soil matrices.

### 4.3 Functional Type and Density‐Dependent Regulation

Our study underscores the role of earthworm functional type in shaping microbial dynamics and soil structure, with important implications for SOC stabilization. This functional divergence was evident across earthworm types: as epigeic earthworms increased microbial biomass carbon, whereas endogeic earthworms increased both aggregate stability and macro‐aggregate formation (Figure 6). Endogeic earthworms create dense, semi‐permanent burrow networks and produce casts rich in binding agents, traits fundamental for forming and stabilizing macro‐aggregates (Knowles et al. 2016). The reduction in MBC associated with endogeic earthworms (Figure 6) can be attributed to direct microbial consumption and resource competition (Blouin et al. 2025; Zou 2025). More importantly, the intense priming effect induced by gut passage and mucus secretion (Mummey et al. 2006; Bernard et al. 2012) not only accelerates microbial turnover but also transforms organic matter. Crucially, the products of this accelerated microbial metabolism were not entirely lost as CO_2_ but were repurposed into key stabilizing agents for soil aggregates, setting the stage for long‐term SOC sequestration.

The resulting soil aggregation was further modulated by earthworm density. Earthworm density was positively correlated with the proportion of macro‐aggregates (*p* < 0.001) but negatively correlated with micro‐aggregates (*p* < 0.001; Figure S10), indicating density‐dependent impacts on soil aggregation dynamics.

Thus, earthworm functional contrasts align with a broader “fast–slow” carbon cycling paradigm: epigeic species enhance rapid carbon turnover by increasing microbial biomass and activity, whereas endogeic species contribute to long‐term carbon stabilization through physical protection mechanisms such as aggregate formation and MAOC accumulation. Carbon from the transient microbial pool is efficiently translocated and incorporated into the physical architecture of macro‐aggregates, where it becomes occluded and protected (Angst et al. 2022, 2024). By diverting carbon from decomposition into aggregates, endogeic earthworms act as key biogeochemical engineers that establish a sustainable pathway for long‐term carbon stabilization. These results highlight the essential role of earthworm functional type in simultaneously enhancing soil structure and promoting carbon storage (Wu et al. 2017).

## 5 Conclusion

Our global meta‐analysis demonstrates that earthworms can act as net enhancers of SOC and provides a mechanistic explanation for the seemingly contradictory results reported in the literature by showing that their net effect is a function of carbon input and time. This net effect arises from the preferential accumulation of carbon in mineral‐associated pools (MAOC), rather than particulate forms (POC). This outcome is fundamentally governed by carbon supply: under sustained plant‐derived inputs, earthworms promote a microbial pathway that increases carbon use efficiency and channels carbon into stable aggregates without increasing metabolic losses. Functional variation among earthworms further modulates this trajectory, with endogeic species most notably driving the physical occlusion of microbial residues within macro‐aggregates, establishing a durable stabilization pathway.

Together, these findings recast earthworms as organisms that simultaneously mediate mineralization and stabilization, and as context‐dependent ecosystem engineers whose net effect depends on carbon availability and time. While this meta‐analysis demonstrates their capacity to enhance soil carbon under sustained inputs, we acknowledge that in environments with fluctuating or reduced carbon supply (e.g., due to agricultural practices, climate disturbances, or deforestation), the balance may shift toward mineralization. This represents an important area for future investigation. Nevertheless, despite uncertainties regarding biome representation, experimental settings, and temporal extrapolation, our results underscore the urgency of incorporating earthworm functional traits and carbon‐input dynamics into Earth system models and climate‐smart soil management strategies. By identifying the conditions under which earthworms transition from short‐term mineralizers to long‐term enhancers, this study fundamentally reshapes their role in the global carbon cycle and elevates them as critical agents in nature‐based climate solutions.

## Author Contributions

H.R., H.Y.H.C., X.Z., and P.B.R. designed the research; Y.L., Y.F., J.N., and T.R. collected the data; J.L., H.Y.H.C., and Y.L. performed the analysis; Y.L. and J.L. wrote the first draft of the manuscript; H.Y.H.C., H.R., X.Z., P.B.R., G.W., and J.C. critically commented on the manuscript and added and/or revised content. All authors approved the final manuscript.

## Funding

This study was supported by the National Key Research and Development Program of China (No. 2021YFD2200403, 2023YFD2200404); the National Natural Science Foundation of China (Grant No. 32101339, 32071594, and 32071832); and the Key Subject of Ecology of Jiangsu Province (SUJIAOYANHAN[2022]No.2).

## Ethics Statement

This presented research did not involve human subjects or animals that would require ethics approval. All methods were carried out in compliance with local and national regulations.

## Conflicts of Interest

The authors declare no conflicts of interest.

## Supporting information


**Data S1:** gcb70815‐sup‐0001‐supinfo.docx.

## Data Availability

The data and R code used can be found in Figshare at https://doi.org/10.6084/m9.figshare.30416218.
