# Supplementary material for: Earthworms Enhance Global Soil Carbon Storage Through Microbial–Mineral Stabilization
Source: Glob Chang Biol. 2026 Mar 19;32(3):e70815. doi: 10.1111/gcb.70815 (PMC13001015; doi:10.1111/gcb.70815)
Supplement: Supplementary file 1 — Data S1: gcb70815‐sup‐0001‐supinfo.docx. [file GCB-32-e70815-s001.docx]

**Supporting Information for**

**Earthworms enhance global soil carbon storage through microbial–mineral stabilization**

Yuanyuan Li^1, 2#^, Jiahui Liao^1#^, Peter B. Reich^3, 4^, Yu Fang^1^, Jiajie Cao^1^, Juanping Ni^1^, Tingting Ren^1^, Guobing Wang^1^, Xiaoming Zou^5^*, Honghua Ruan^1^*, Han Y.H. Chen^6, 7, 3^*

^1^ Joint Center for Sustainable Forestry in Southern China, Nanjing Forestry University; Nanjing 210037, P.R. China

^2^ School of Food Science, Nanjing Xiaozhuang University; Nanjing 211171, P.R. China

^3^ Institute for Global Change Biology, School for Environment and Sustainability, University of Michigan; Ann Arbor, MI, USA.

^4^ Department of forest resources, 1530 Cleveland Ave. N., University of Minnesota; St. Paul, MN 55108

^5^ Institute of Agricultural Resources and Environment, Jiangsu Academy of Agricultural Sciences; Nanjing, 210014, China

^6^ College of Grassland Science, Inner Mongolia Agricultural University, Hohhot, 010011, China

^7^ Faculty of Natural Resources Management, Lakehead University; 955 Oliver Road, Thunder Bay, ON P7B 5E1, Canada

^#^These authors contributed equally to this work and should be considered co-first authors

*Corresponding authors: [han.yh.chen@gmail.com](mailto:han.yh.chen@gmail.com)；[xzou2011@gmail.com](mailto:xzou2011@gmail.com)；[hhruan@njfu.edu.cn](mailto:hhruan@njfu.edu.cn)

**This file includes:**

Figures S1 to S10

Table S1 to S2

### Fig. S1. Geographic locations of the studies included in the meta-analysis.


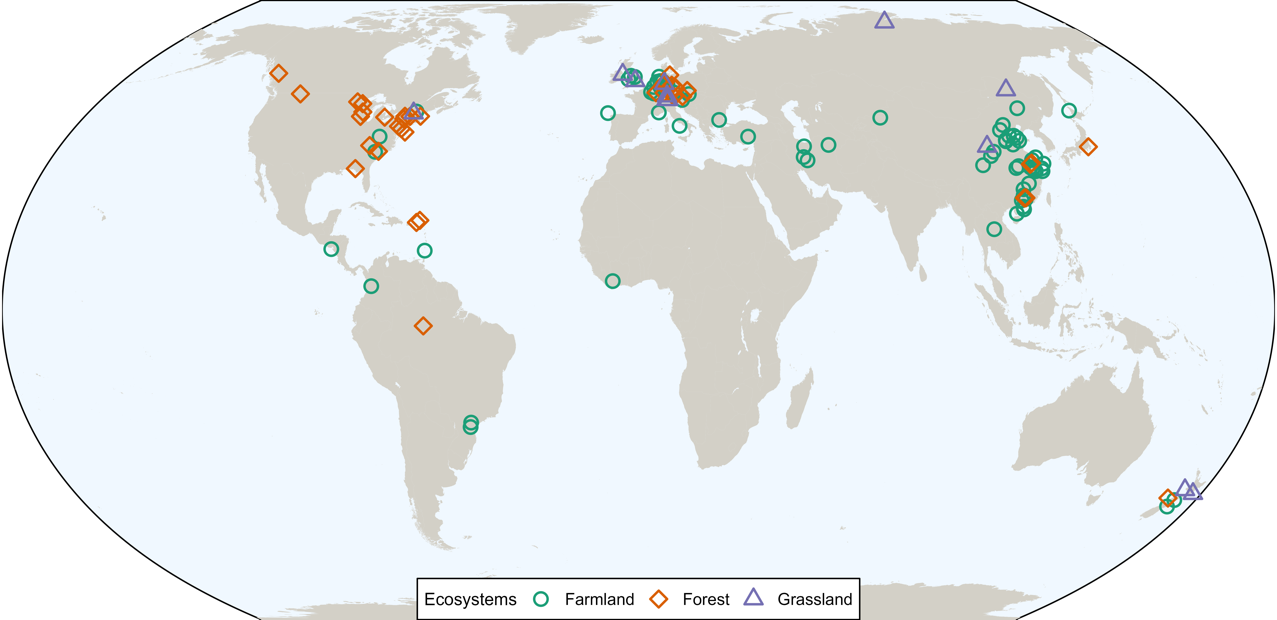


### Fig. S2. Data collection for this meta-analysis. PRISMA diagram showing the process of locating studies included in the dataset of this study.


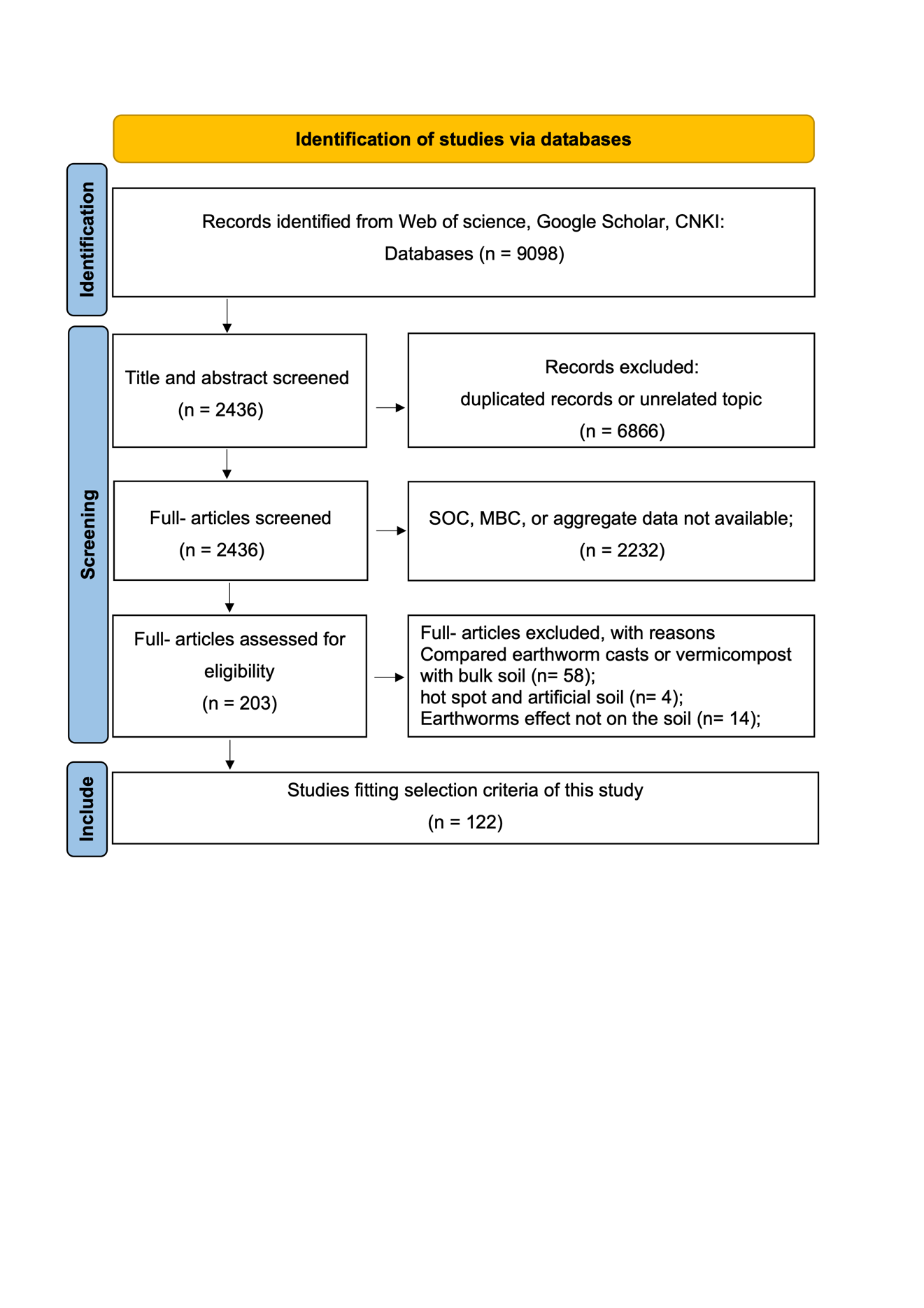


Fig. S3. **Comparison of pooled effect sizes and heterogeneity between the Full Data method and the Complete SD method for SOC**. (a) Forest plot of pooled effect sizes (lnRR) and 95% confidence intervals (CIs) from multilevel meta-analyses. The blue represents the result from the Full Data method (n = 258), and the light blue represents the result from the Complete SD method (n = 230). The vertical dashed line indicates the null effect (lnRR = 0). The Z-score and p-value for the comparison between the two estimates are annotated. (b) Bar chart comparing the heterogeneity (I²) between the two methods.

### Fig. S4. Funnel plot for publication bias assessment for SOC.

Data points (green circles) represent individual effect sizes plotted against precision. The dashed blue line indicates the expected distribution under no bias (Egger's line). Shaded areas indicate confidence intervals around the pooled effect: light blue (90% CI), light green (95% CI), and medium green (99% CI). Egger's test indicates no significant bias (p = 0.315)


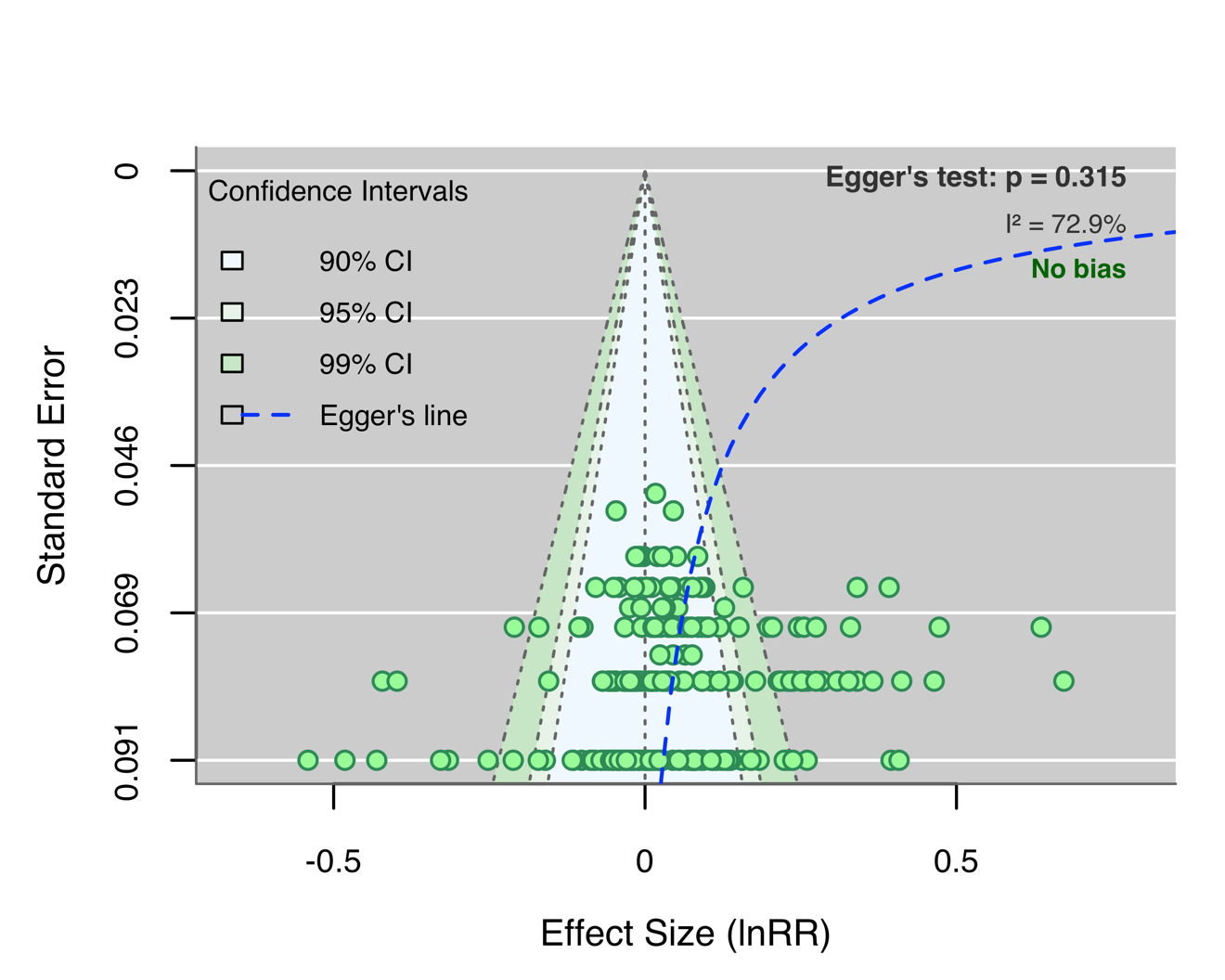


### Fig. S5. Sensitivity analysis of soil organic carbon (SOC) using leave-one-study-out approach.

(a) Leave-one-out analysis: each green point represents the model estimate after omitting one study. The solid red line indicates the overall mean effect size (0.054) and dashed red lines its 95% confidence interval. Effect sizes were stable (range: 0.05-0.06, Robustness index: 0.22) with no influential studies detected. (b) Distribution comparison: green violin plots show individual study effect sizes, and blue violin plots show leave-one-out (LOO) estimates. The close overlap between distributions confirms high robustness of results.


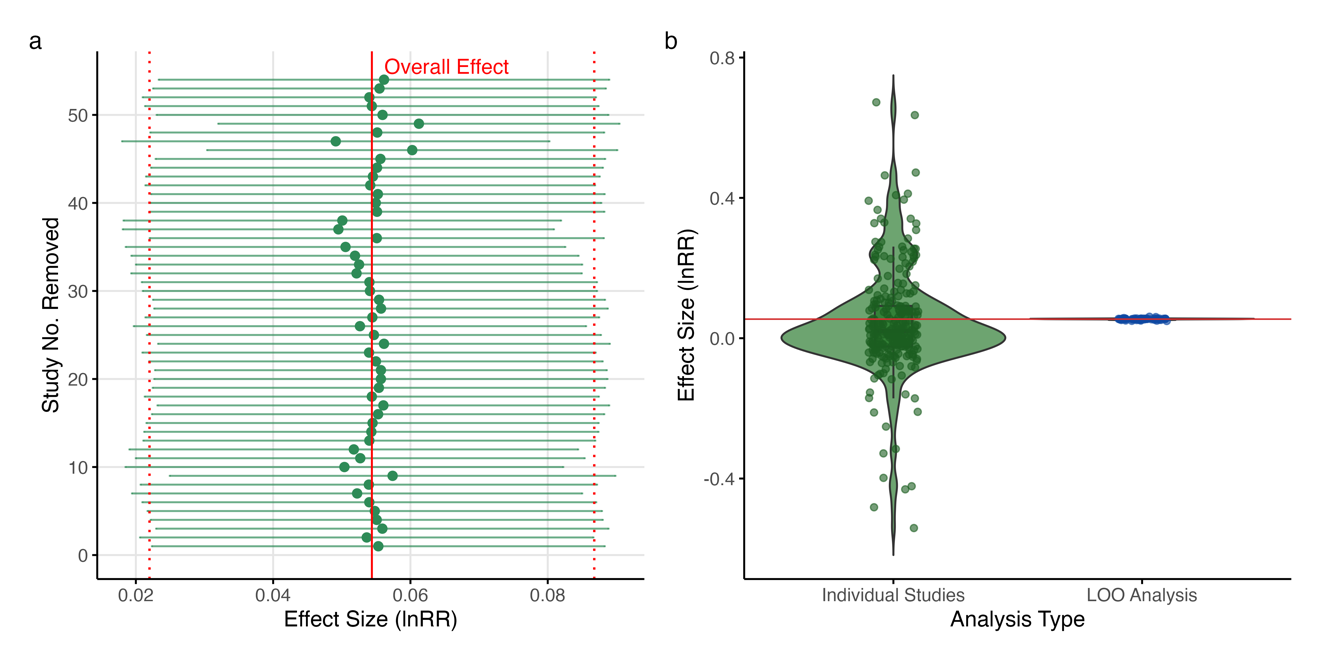


### Fig. S6. Sensitivity analysis using the leave-one-study-out approach.

Leave-one-out analysis, each green point represents the model estimate after omitting one study. The solid red line indicates the overall mean effect size and dashed red lines its 95% confidence interval. (a) POC effect sizes were stable (range: 0.009– 0.114), (b) MAOC effect sizes were stable (range: 0.154– 0.251), (c) microbial basal respiration effect sizes were stable (range: 0.214– 0.293), (d) microbial metabolic quotient (*q*CO₂) (range: 0.129– 0.184), (e) microbial biomass effect sizes were stable (range: 0.054– 0.074), (f) macro-aggregate mean weight diameter effect sizes were stable (range: 0.124– 0.19), (g) macro-aggregate effect sizes were stable (range: 0.093– 0.159), (h) micro-aggregate effect sizes were stable (range: –0.243– –0.174). No influential studies were detected in any of the analyses. Egger’s test revealed no significant publication bias for any variable (p-values for all panels a–h: 0.08, 0.31, 0.87, 0.88, 0.77, 0.39, 0.87, 0.63, respectively)


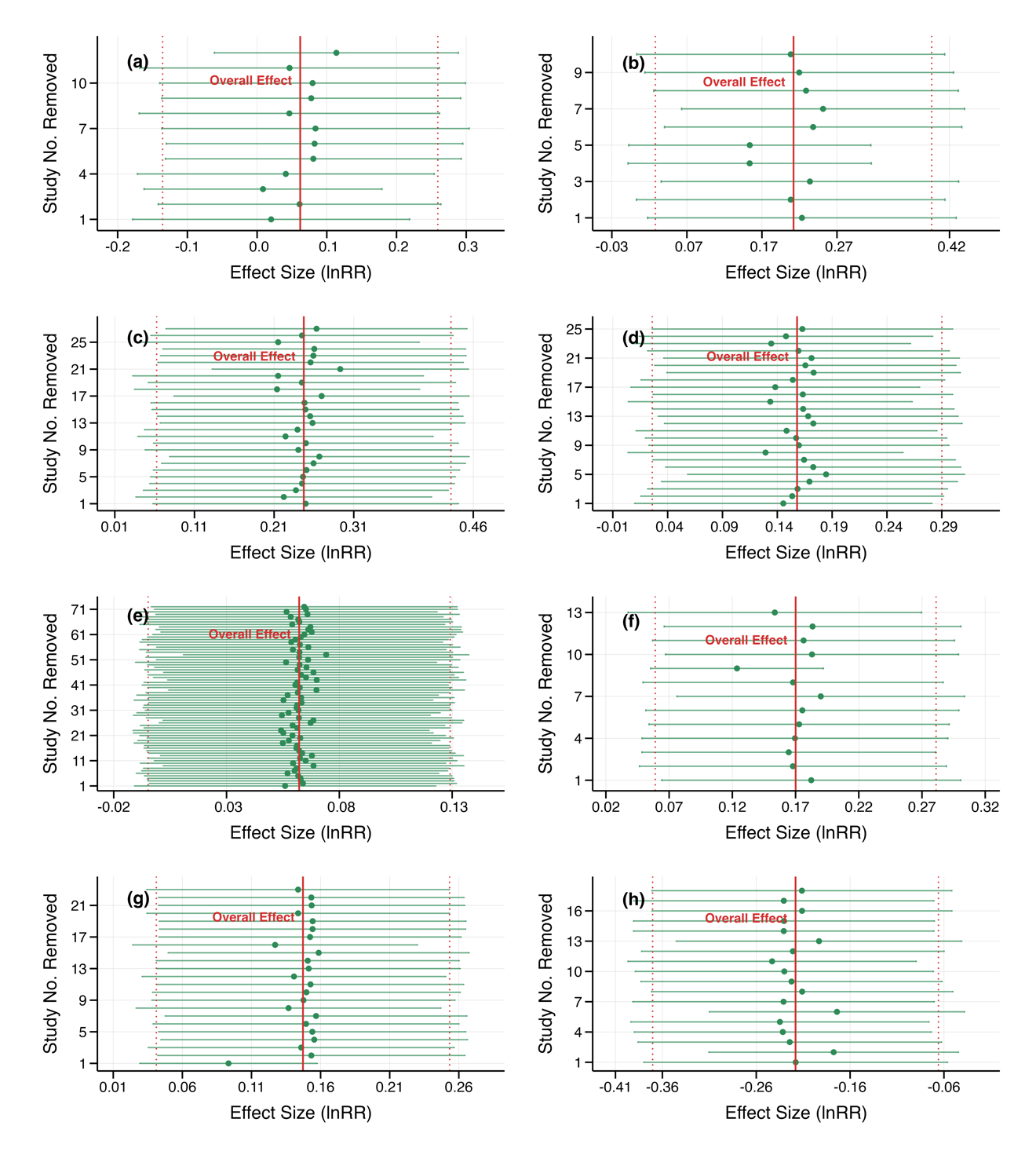


### Fig. S7. Effects of earthworms on soil properties.

Soil pH, ammonium nitrogen (NH_4_^+^-N), nitrate nitrogen (NO_3_^-^ -N), dissolved inorganic nitrogen (DIN), dissolved organic carbon (DOC), Gram+: Gram– bacteria ratio (G+: G- ratio), bacterial biomass, and fungal biomass. Values are mean ± 95% confidence intervals of the percentage effects between the earthworm and control treatments. Each dot represents an observation, with horizontal position indicating its value and vertical stacking reflecting density. The number of observations is beside each attribute without parentheses, and the number of studies is in parentheses.


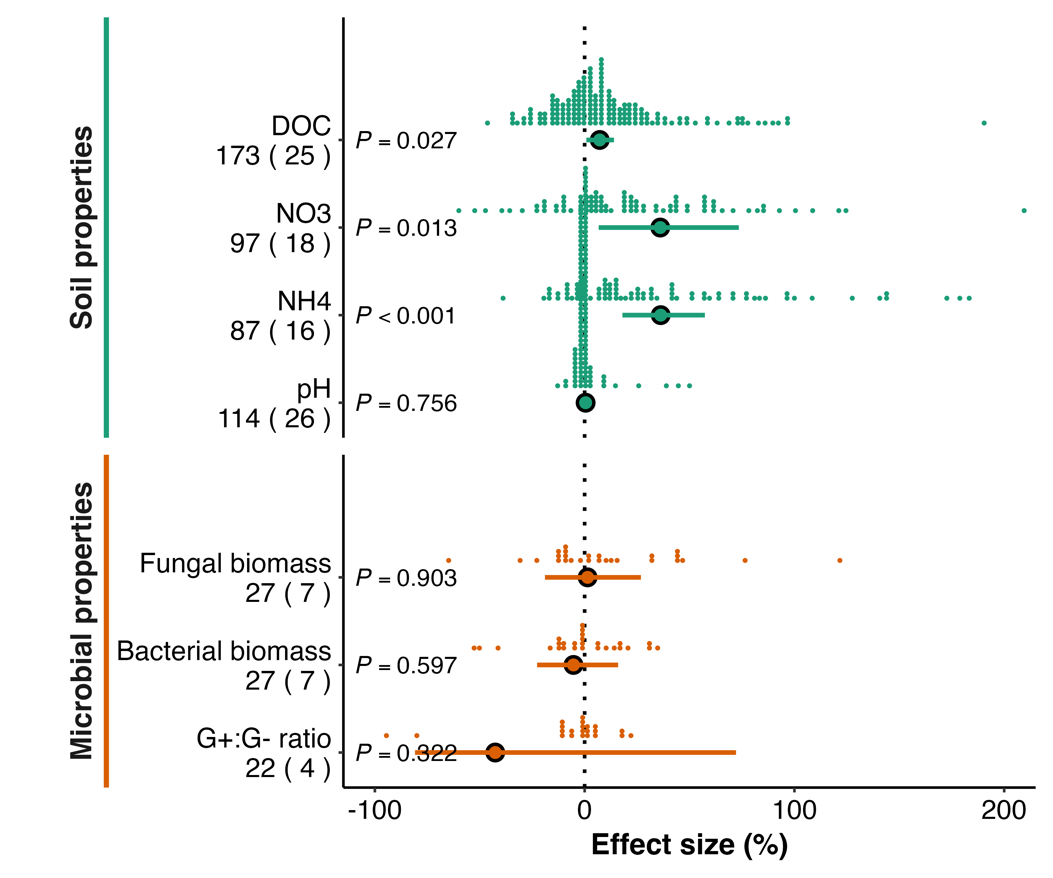


### Fig.S8. Bivariate relationships of SOC with experiment duration among plant types. The figure shows meta‑regression fits between the log‑transformed experimental duration (ED, in days) and the log response ratios (lnRR) of SOC for experiments with earthworm inoculation under different organic input regimes: (a) Plant (live plant-derived input), (b) OM(detrital organic matter input: litter or amendments), and (c) NO (no carbon input). Each filled circle represents an observation, scaled by its inverse‑variance weight (W = 1/V) in the meta‑analysis. Solid (p < 0.05) and dashed (p ≥ 0.05) lines indicate the fitted meta‑regression relationships. The regression slope (β), 95% confidence interval (CI), and p‑value (p) are annotated in each panel.

**
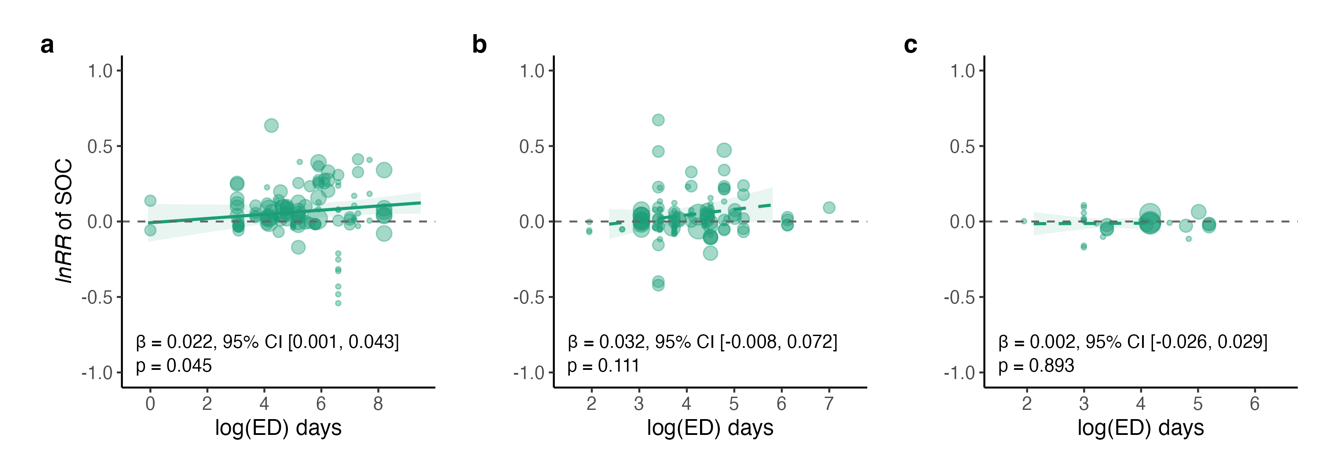
**

### Fig. S9. ****Earthworm effects on FB and micro-aggregate depend on plant input type**.** Responses of fungal-to-bacterial (F:B) ratio and micro-aggregate are shown across different plant input types (Plant: living plant input, OM: detrital input including litter/amendments, and NO: no input). Values represent the mean ± 95% confidence intervals of percentage changes between earthworm and control treatments. Each dot represents a single observation, with horizontal position indicating the effect size and vertical stacking reflecting observation density. The number of observations is indicated beside each attribute (without parentheses), and the number of contributing studies is shown in parentheses.

**
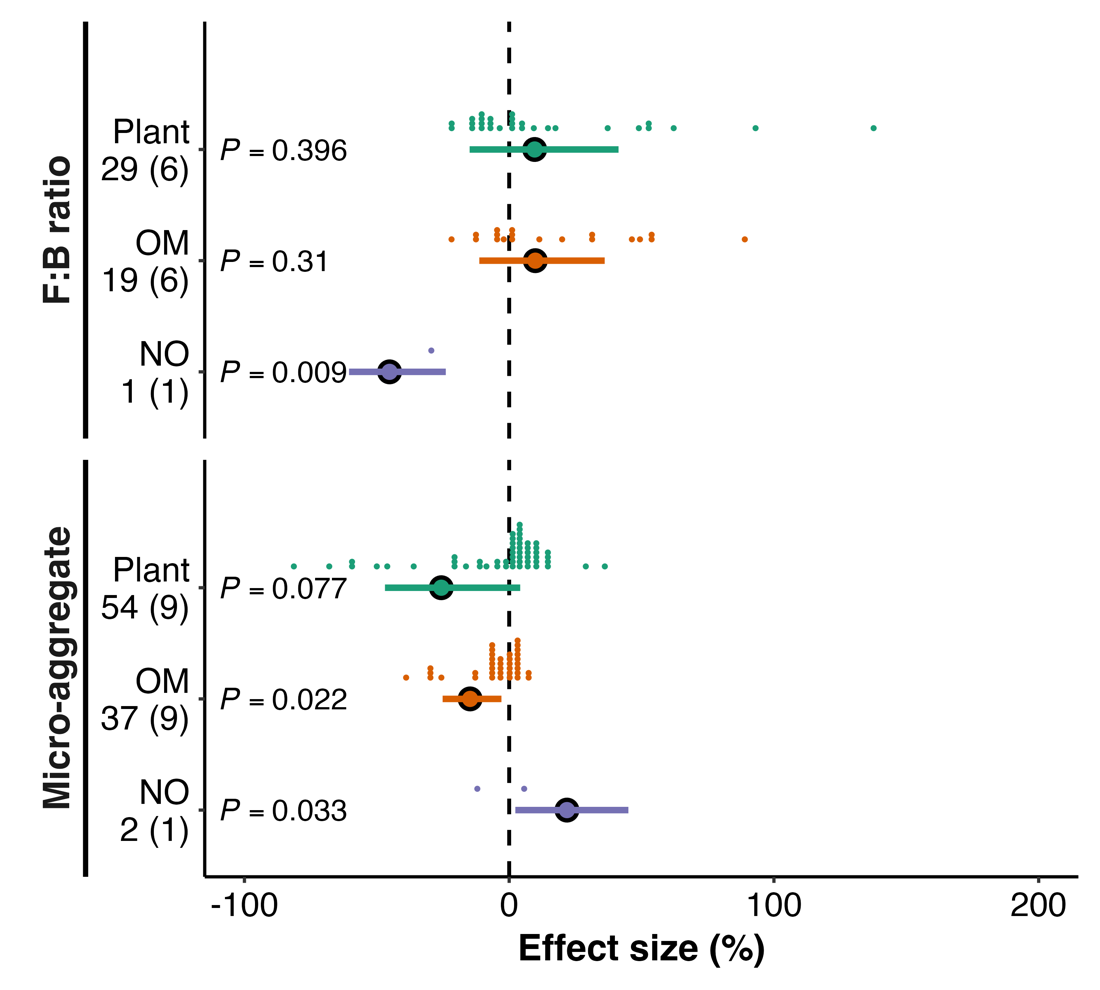
**

### Fig. S10. Bivariate relationships of aggregate fractions and microbial biomass with earthworm density and climate.

Log response ratios (lnRR) of (a) small macro-aggregate and (b) micro-aggregate in response to earthworm density (D, individuals.m^-2^), and (c) microbial biomass carbon in response to mean annual temperature (℃) across all studies with earthworm inoculation. Each filled circle represents an observation, scaled by its inverse‑variance weight (W = 1/V) in the meta‑analysis. Solid lines indicate the predicted meta‑regression fit. The regression slope (β) and its corresponding p‑value (p).


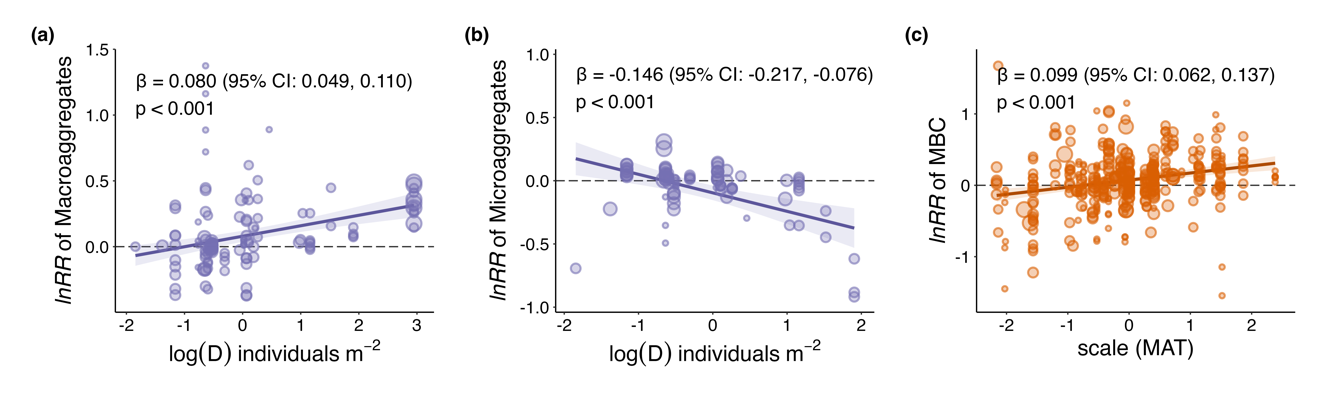


### Table S1. List of the 122 papers used in this meta-analysis.

| NO. | uni.ID | Publication |
| --- | --- | --- |
| 1 | 4 | Dynamics of soil N2O emissions and functional gene abundance in response to biochar application in the presence of earthworms. DOI:10.1016/j.envpol.2020.115670 |
| 2 | 12 | Effect of decomposition products produced in the presence or absence of epigenic earthworms and minerals on soil carbon stabilization. DOI:10.1016/j.soilbio.2021.108308 |
| 3 | 13 | The geophagous earthworm Metaphire guillelmi e ects on rhizosphere microbial community structure and functioning vary with plant species.DOI:10.1016/j.geoderma.2020.114647 |
| 4 | 17 | Assessment of earthworm activity on Cu, Cd, Pb and Zn bioavailability in contaminated soils using biota to soil accumulation factor and DTPA extraction.DOI:10.1016/j.ecoenv.2020.110513 |
| 5 | 18 | Earthworms accelerate rice straw decomposition and maintenance of soil organic carbon dynamics in rice agroecosystems.DOI:10.7717/peerj.9870 |
| 6 | 20 | Legacy Effects of Agricultural Practices Override Earthworm Control on C Dynamics in Kiwifruit Orchards.DOI:10.3389/fenvs.2020.545609 |
| 7 | 23 | Long-term e ects of earthworms (Lumbricus rubellus Ho meister, 1843) on activity and composition of soil microbial community under laboratory conditions.DOI:10.1016/j.apsoil.2019.103463 |
| 8 | 24 | Earthworms negate the adverse effect of arbuscular mycorrhizae on living bacterial biomass and bacterial necromass accumulation in a subtropical soil.DOI:10.1016/j.soilbio.2020.108052 |
| 9 | 25 | The interactions between biochar and earthworms, and their influence on soil properties and clover growth: A 6-month mesocosm experiment.DOI:10.1016/j.apsoil.2019.103402 |
| 10 | 27 | Nitrogen fertilization alters the effects of earthworms on soil physicochemical properties and bacterial community structure.DOI:10.1016/j.apsoil.2019.103478 |
| 11 | 32 | Earthworms promote the accumulation of maize root-derived carbon in a black soil of Northeast China, especially in soil from long-term no-till.DOI:10.1016/j.geoderma.2019.01.003 |
| 12 | 33 | Earthworms suppress thrips attack on tomato plants by concomitantly modulating soil properties and plant chemistry.DOI:10.1016/j.soilbio.2018.11.023 |
| 13 | 35 | Resilience of soil functions to transient and persistent stresses is improved more by residue incorporation than the activity of earthworms.DOI:10.1016/j.apsoil.2019.03.008 |
| 14 | 38 | The Role of Diﬀerent Earthworm Species (Metaphire Hilgendorfi and Eisenia Fetida) on CO2 Emissions and Microbial Biomass during Barley Decomposition.DOI:10.3390/su11236544 |
| 15 | 39 | Temporal Variation of Earthworm Impacts on Soil Organic Carbon under Diﬀerent Tillage Systems.DOI:10.3390/ijerph16111908 |
| 16 | 41 | Litter chemistry in uences earthworm e ects on soil carbon loss and microbial carbon acquisition.DOI:10.1016/j.soilbio.2018.05.012 |
| 17 | 45 | Responses of Saline Soil Properties and Cotton Growth to Diﬀerent Organic Amendments.DOI:10.1016/S1002-0160(17)60464-8 |
| 18 | 47 | Similar positive e ects of bene cial bacteria, nematodes and earthworms on soil quality and productivity.DOI:10.1016/j.apsoil.2018.06.016 |
| 19 | 49 | Cooperation of earthworm and arbuscular mycorrhizae enhanced plant N uptake by balancing absorption and supply of ammonia.DOI:10.1016/j.soilbio.2017.10.038 |
| 20 | 50 | The e ect of earthworm and arbuscular mycorrhizal fungi on availability and chemical distribution of Zn, Fe and Mn in a calcareous soil.DOI:10.1016/j.apsoil.2018.06.002 |
| 21 | 51 | Differential effects of two earthworm species on Fusarium wilt of strawberry.DOI:10.1016/j.apsoil.2018.02.024 |
| 22 | 53 | Seabird guano and phosphorus fractionation in a rhizosphere with earthworms.DOI:10.1016/j.apsoil.2017.08.006 |
| 23 | 54 | Earthworms and phosphate-solubilizing bacteria enhance carbon accumulation in manure-amended soils.DOI:10.1007/s11368-016-1482-6 |
| 24 | 58 | Impacts of Endemic Maoridrilus Earthworms (Megascolecidae) in Biosolids-Amended Soil.DOI:10.2134/jeq2016.06.0207 |
| 25 | 59 | Earthworms and root-knot nematodes: effect on soil biological activity and tomato growth.DOI:10.5433/1679-0359.2017v38n4Supl1p2449 |
| 26 | 60 | Amynthas agrestis invasion increases microbial biomass in Mid-Atlantic deciduous forests.DOI:10.1016/j.soilbio.2017.07.018 |
| 27 | 62 | Earthworms (Amynthas spp.) increase common bean growth, microbial biomass, and soil respiration.DOI:10.5433/1679-0359.2017v38n5p2887 |
| 28 | 64 | Six months of L. terrestris L. activity in root-formed biopores increases nutrient availability, microbial biomass and enzyme activity.DOI:10.1016/j.apsoil.2017.08.015 |
| 29 | 65 | Effect of earthworms and arbuscular mycorrhizal fungi on the microbial community and maize growth under salt stress.DOI:10.1016/j.apsoil.2016.06.005 |
| 30 | 66 | Pesticide seed dressings can affect the activity of various soil organisms and reduce decomposition of plant material.DOI:10.1186/s12898-016-0092-x |
| 31 | 68 | Sewage sludge application strongly modi es earthworm impact on microbial and biochemical attributes in a semi-arid calcareous soil from Iran.DOI:10.1016/j.apsoil.2015.11.022 |
| 32 | 69 | Plants modify the effects of earthworms on the soil microbial community and its activity in a subtropical ecosystem.DOI:10.1016/j.soilbio.2016.09.020 |
| 33 | 70 | Enhancing pentachlorophenol degradation by vermicomposting associated bioremediation.DOI:10.1016/j.ecoleng.2015.12.004 |
| 34 | 71 | Resource Utilization by Native and Invasive Earthworms and Their Effects on Soil Carbon and Nitrogen Dynamics in Puerto Rican Soils.DOI:10.3390/f7110277 |
| 35 | 75 | Earthworm ecosystem service and dis-service in an N-enriched agroecosystem: Increase of plant production leads to no effects on yield-scaled N2O emissions.DOI:10.1016/j.soilbio.2014.12.009 |
| 36 | 77 | The Effect of Biochar and Its Interaction with the Earthworm Pontoscolex corethrurus on Soil Microbial Community Structure in Tropical Soils.DOI:10.1371/journal.pone.0124891 |
| 37 | 78 | Soil properties and maize growth in saline and nonsaline soils using cassava-industrial waste compost and vermicompost with or without earthworms.DOI:10.1002/ldr.2208 |
| 38 | 79 | Additive effects of earthworms, nitrogen-rich litter and elevated soil temperature on N2O emission and nitrate leaching from an arable soil.DOI:10.1016/j.apsoil.2014.10.006 |
| 39 | 81 | Interaction between earthworms and arbuscular mycorrhizal fungi on the degradation of oxytetracycline in soils.DOI:10.1016/j.soilbio.2015.08.020 |
| 40 | 82 | Effects of addition of maize litter and earthworms on C mineralization and aggregate formation in single and mixed soils differing in soil organic carbon and clay content.DOI:10.1016/j.pedobi.2014.03.001 |
| 41 | 84 | The effect of earthworms (Lumbricus rubellus) and simulated tillage on soil organic carbon in a long-term microcosm experiment.DOI:10.1016/j.soilbio.2014.07.011 |
| 42 | 86 | Effects of biochar, earthworms, and litter addition on soil microbial activity and abundance in a temperate agricultural soil.DOI:10.1007/s00374-014-0968-x |
| 43 | 87 | The combined effects of earthworms and arbuscular mycorrhizal fungi on microbial biomass and enzyme activities in a calcareous soil spiked with cadmium.DOI:10.1016/j.apsoil.2013.10.006 |
| 44 | 88 | Dose-dependent reactions of Aporrectodea caliginosa to per uorooctanoic acid and per uorooctanesulfonic acid in soil.DOI:10.1016/j.ecoenv.2013.05.012 |
| 45 | 92 | Inoculating maize fields with earthworms (Aporrectodea trapezoides) and an arbuscular mycorrhizal fungus (Rhizophagus intraradices) improves mycorrhizal community structure and increases plant nutrient uptake.DOI:10.1007/s00374-013-0815-5 |
| 46 | 93 | Eﬀects of Earthworms and Ryegrass on the Removal of Fluoranthene from Soil.DOI:10.1016/S1002-0160(13)60045-4 |
| 47 | 94 | Interactions between earthworms and residues of differing quality affecting aggregate stability and microbial dynamics.DOI:10.1016/j.apsoil.2012.10.008 |
| 48 | 96 | Eﬀects of Epigeic Earthworms on Decomposition of Wheat Straw and Nutrient Cycling in Agricultural Soils in a Reclaimed Salinity Area: A Microcosm Study.DOI:10.1016/S1002-0160(12)60058-7 |
| 49 | 98 | Earthworms Reduce the Abundance of Nematodes and Enchytraeids in a Soil Mesocosm Experiment Despite Abundant Food Resources.DOI:10.2136/sssaj2011.0035 |
| 50 | 99 | A microcosm study of the common night crawler earthworm (Lumbricus terrestris) and physical, chemical and biological properties of a designed urban soil.DOI:10.1007/s11252-010-0145-4 |
| 51 | 101 | Belowground interactive effects of elevated CO2, plant diversity and earthworms in grassland microcosms.DOI:10.1016/j.baae.2011.08.004 |
| 52 | 103 | The wave towards a new steady state: effects of earthworm invasion on soil microbial functions.DOI:10.1007/s10530-011-0053-4 |
| 53 | 104 | Soil CO2 flux affected by Aporrectodea caliginosa earthworms.DOI:10.2478/s11535-010-0017-1 |
| 54 | 105 | Effects of Aporrectodea caliginosa (Savigny) on nitrogen mobilization and decomposition of elevated-CO2 Charlock mustard litter.DOI:10.1002/jpln.201000092 |
| 55 | 106 | Effects of mixed cropping, earthworms (Pheretima sp.), and arbuscular mycorrhizal fungi (Glomus mosseae) on plant yield, mycorrhizal colonization rate, soil microbial biomass, and nitrogenase activity of free-living rhizosphere bacteria.DOI:10.1016/j.pedobi.2008.10.004 |
| 56 | 109 | Decomposition and mineralization of energy crop residues governed by earthworms.DOI:10.1016/j.soilbio.2009.04.015 |
| 57 | 111 | Earthworm (Metaphire guillelmi) effects on rice photosynthates distribution in the plant–soil system.DOI:10.1007/s00374-007-0250-6 |
| 58 | 112 | C and N turnover of fermented residues from biogas plants in soil in the presence of three different earthworm species (Lumbricus terrestris, Aporrectodea longa, Aporrectodea caliginosa).DOI:10.1016/j.soilbio.2007.12.026 |
| 59 | 118 | The effect of litter quality and soil faunal composition on organic matter dynamics in post-mining soil: A laboratory study.DOI:10.1016/j.apsoil.2007.04.001 |
| 60 | 119 | Invasion of a deciduous forest by earthworms: Changes in soil chemistry, microflora, microarthropods and vegetation.DOI:10.1016/j.soilbio.2006.12.019 |
| 61 | 121 | Endogeic earthworms alter carbon translocation by fungi at the soil–litter interface.DOI:10.1016/j.soilbio.2007.05.028 |
| 62 | 124 | Effects of macro-decomposers on litter decomposition and soil properties in alpine pastureland: A mesocosm experiment.DOI:10.1016/j.apsoil.2006.02.004 |
| 63 | 125 | Effect of earthworm addition on soil nitrogen availability, microbial biomass and litter decomposition in mesocosms.DOI:10.1007/s00374-003-0696-0 |
| 64 | 126 | Effect of Aporrectodea trapezoides activity on seedling growth of Pseudotsuga menziesii, nutrient dynamics and microbial activity in different forest soils.DOI:10.1016/S0378-1127(02)00010-5 |
| 65 | 130 | Effect of Eisenia foetida earthworms on mineralization kinetics, microbial biomass, enzyme activities, respiration and labile C fractions of three soils treated with a composted organic residue.DOI:10.1007/s00374-003-0612-7 |
| 66 | 134 | Exotic Earthworms Accelerate Plant Litter Decomposition in a Puerto Rican Pasture and a Wet Forest.DOI:10.1890/1051-0761(2002)012[1406:EEAPLD]2.0.CO;2 |
| 67 | 135 | Influence of earthworm invasion on soil microbial biomass and activity in a northern hardwood forest.DOI:10.1016/S0038-0717(02)00210-9 |
| 68 | 137 | Interaction of the earthworm Diplocardia mississippiensis (Megascolecidae) with microbial and nutrient dynamics in a subtropical Spodosol.DOI:10.1016/S0038-0717(01)00049-9 |
| 69 | 138 | Earthworms indirectly reduce the effects of take-all (Gaeumannomyces graminis var. tritici) on soft white spring wheat Triticum aestivum cv. Fielder).DOI:10.1016/s0038-0717(01)00071-2 |
| 70 | 139 | Different behavioral patterns of the earthworms Octolasion tyrtaeum and Diplocardia spp. in tallgrass prairie soils: potential influences on plant growth.DOI:10.1007/s003740100370 |
| 71 | 140 | Lumbricus terrestris in a soil core experiment: nutrient-enrichment processes (NEP) and gut-associated processes (GAP) and their effect on microbial biomass and microbial activity.DOI:10.1016/0038-0717(95)00090-2 |
| 72 | 141 | Earthworms facilitate stabilization of both more-available maize biomass and more-recalcitrant maize biochar on mineral particles in an agricultural soil.DOI:10.1016/j.soilbio.2023.109278 |
| 73 | 142 | Individual and combined effects of earthworms and Sphingobacterium sp. on soil organic C, N forms and enzyme activities in non-contaminated and Cd-contaminated soil.DOI:10.1016/j.ejsobi.2023.103576 |
| 74 | 143 | Impacts of earthworms and their excreta on peat soil properties and bacterial community.DOI:10.1016/j.gecco.2023.e02594 |
| 75 | 144 | Earthworm activity effectively mitigated the negative impact of microplastics on maize growth.DOI:10.1016/j.jhazmat.2023.132121 |
| 76 | 145 | Earthworms and long-term straw management practices interactively affect soil carbon and nitrogen forms across soil depths.DOI:10.1016/j.ejsobi.2023.103478 |
| 77 | 146 | Soil fauna alter the responses of greenhouse gas emissions to changes in water and nitrogen availability.DOI:10.1016/j.soilbio.2023.108990 |
| 78 | 147 | Effects of Earthworms and Phosphate-Solubilizing Bacteria on Carbon Sequestration in Soils Amended with Manure and Slurry: A 4-Year Field Study.DOI:10.3390/agronomy12092064 |
| 79 | 149 | Roxarsone reduces earthworm-mediated nutrient cycling by suppressing aggregate formation and enzymic activity in soil with manure application.DOI:10.1016/j.envpol.2024.124777 |
| 80 | 150 | Earthworm co-invasion by Amynthas tokioensis and Amynthas agrestis affects soil microaggregate bacterial communities.DOI:10.1016/j.apsoil.2023.105224 |
| 81 | 152 | Earthworm influence on soil aggregate distribution and protected carbon at managed forest sites in Vermont, USA.DOI:10.1016/j.soilbio.2024.109534 |
| 82 | 153 | Effects of earthworms on microbial community structure, functionality and soil properties in soil cover treatments for mine tailings rehabilitation.DOI:10.1016/j.ejsobi.2024.103603 |
| 83 | 154 | The effects of roots and earthworms on aggregate size distribution and their associated carbon under contrasting soil types and soil moisture conditions.DOI:10.1016/j.catena.2024.108434 |
| 84 | 155 | Soil structure shifts with earthworms under different organic fertilization in salt-affected soils.DOI:10.1002/ldr.4966 |
| 85 | 159 | Earthworms increase nitrogen uptake by lettuce and change short-term soil nitrogen dynamics.DOI:10.1016/j.apsoil.2022.104488 |
| 86 | 160 | Earthworm (Pheretima guillelmi)-mycorrhizal fungi (Funneliformis mosseae) association mediates rhizosphere responses in white clover.DOI:10.1016/j.apsoil.2021.104371 |
| 87 | 161 | Soil aggregate organic carbon and clover root characteristics as affected by earthworms (Metaphire guillelmi).DOI:10.1002/ldr.4410 |
| 88 | 162 | Synergistic effects of straw and earthworm addition on microbial diversity and microbial nutrient limitation in a subtropical conservation farming system.DOI:10.1016/j.still.2022.105500 |
| 89 | 163 | Plants and earthworms control soil carbon and water quality trade-offs in turfgrass mesocosms.DOI:10.1016/j.scitotenv.2020.141884 |
| 90 | 165 | Impacts induced by the combination of earthworms, residue and tillage on soil organic carbon dynamics using 13C labelling technique and X-ray computed tomography.DOI:10.1016/j.still.2020.104737 |
| 91 | 166 | Impact of different earthworm ecotypes on water stable aggregates and soil water holding capacity.DOI:10.1007/s00374-020-01432-5 |
| 92 | 167 | Effect of earthworms on soil physico-hydraulic and chemical properties, herbage production, and wheat growth on arable land converted to ley.DOI:10.1016/j.scitotenv.2019.136491 |
| 93 | 168 | Integrated reclamation of saline soil nitrogen transformation in the hyphosphere by earthworms and arbuscular mycorrhizal fungus.DOI:10.1016/j.apsoil.2018.12.005 |
| 94 | 169 | Composition and superposition of alluvial deposits drive macro-biological soil engineering and organic matter dynamics in floodplains.DOI:10.1016/j.geoderma.2019.113899 |
| 95 | 170 | Pioneer plant Phalaris arundinacea and earthworms promote initial soil structure formation despite strong alluvial dynamics in a semi-controlled field experiment.DOI:10.1016/j.catena.2019.04.001 |
| 96 | 171 | Soil structure formation and organic matter distribution as a ected by earthworm species interactions and crop residue placement.DOI:10.1016/j.geoderma.2018.07.033 |
| 97 | 172 | Cooperation between arbuscular mycorrhizal fungi and earthworms promotes the physiological adaptation of maize under a high salt stress.DOI:10.1007/s11104-017-3481-9 |
| 98 | 173 | Impacts of earthworm activity on the fate of straw carbon in soil: a microcosm experiment.DOI:10.1007/s11356-018-1397-4 |
| 99 | 174 | Distribution and fractionation of cadmium in soil aggregates affected by earthworms (Eisenia fetida) and manure compost.DOI:10.1007/s11368-016-1433-2 |
| 100 | 175 | Effect of the endogeic earthworm Aporrectodea tuberculata on aggregation and carbon redistribution in uninvaded forest soil columns.DOI:10.1016/j.soilbio.2016.06.016 |
| 101 | 176 | Species-speci c effects of earthworms on microbial communities and the fate of litter-derived carbon.DOI:10.1016/j.soilbio.2016.06.004 |
| 102 | 177 | Lumbricid earthworm effects on incorporation of root and leaf litter into aggregates in a forest soil, New York State.DOI:10.1007/s10533-015-0126-z |
| 103 | 178 | Aggregate formation and carbon sequestration by earthworms in soil from a temperate forest exposed to elevated atmospheric CO2: A microcosm experiment.DOI:10.1016/j.soilbio.2013.09.023 |
| 104 | 179 | Earthworm effects on the incorporation of litter C and N into soil organic matter in a sugar maple forest.DOI:10.1890/12-1760.1 |
| 105 | 180 | Changes in soil aggregate carbon dynamics under no-tillage with respect to earthworm biomass revealed by radiocarbon analysis.DOI:10.1016/j.still.2012.07.003 |
| 106 | 181 | Functional attributes: Compacting vs decompacting earth-worms and influence on soil structure.DOI:10.1093/czoolo/58.4.556 |
| 107 | 182 | Impacts of invasive Asian (Amynthas hilgendorfi) and European (Lumbricus rubellus) earthworms in a North American temperate deciduous forest.DOI:10.1007/s10530-012-0208-y |
| 108 | 183 | Interactive effects of plants and earthworms on the physical stabilization of soil organic matter in aggregates.DOI:10.1007/s11104-012-1199-2 |
| 109 | 185 | Earthworms and litter management contributions to ecosystem services in a tropical agroforestry system.DOI:10.1890/09-0795.1 |
| 110 | 186 | Earthworm impacts on soil organic matter and fertilizer dynamics in tropical hillside agroecosystems of Honduras.DOI:10.1016/j.pedobi.2010.03.002 |
| 111 | 191 | Interactive effects of functionally different earthworm species on aggregation and incorporation and decomposition of newly added residue carbon.DOI:10.1016/j.geoderma.2005.01.005 |
| 112 | 192 | Protection of soil carbon by microaggregates within earthworm casts.DOI:10.1016/j.soilbio.2004.07.035 |
| 113 | 193 | The respective roles of roots and earthworms in restoring physical properties of Vertisol under a Digitaria decumbens pasture (Martinique, WI).DOI:10.1016/j.agee.2003.12.012 |
| 114 | 194 | Interactions between earthworms (Aporrectodea caliginosa), plants and crop residues for restoring properties of a degraded arable soil.DOI:10.1078/0031-4056-00273 |
| 115 | 195 | The influence of earthworm community structure on the distribution and movement of solutes in a chisel-tilled soil.DOI:10.1016/S0929-1393(02)00063-X |
| 116 | 196 | Subtropical forest macro-decomposers rapidly transfer litter carbon and nitrogen into soil mineral-associated organic matter.DOI:10.1016/j.fecs.2024.100172 |
| 117 | 197 | Earthworms regulate soil microbial and plant residues through decomposition.DOI:10.1016/j.geoderma.2024.117040 |
| 118 | 198 | How the effect of earthworms on soil organic matter mineralization and stabilization is affected by litter quality and stage of soil development.DOI:10.1007/s10533-024-01182-8 |
| 119 | 201 | Earthworms act as biochemical reactors to convert labile plant compounds into stabilized soil microbial necromass.DOI:10.1038/s42003-019-0684-z |
| 120 | 202 | Earthworm-induced N2O emissions in a sandy soil with surface-applied crop residues.DOI:10.1016/j.pedobi.2011.09.005 |
| 121 | 203 | Earthworms increase the ratio of bacteria to fungi in northern hardwood forest soils, primarily by eliminating the organic horizon.DOI:10.1016/j.soilbio.2011.06.017 |
| 122 | 204 | Rapid incorporation of carbon from fresh residues into newly formed stable microaggregates within earthworm casts.DOI:10.1111/j.1365-2389.2004.00603.x |

### Table S2 The results of mixed-effects meta-regression models for soil organic carbon (SOC) by simultaneously examining multiple potential predictors in a full model. The relationships are evaluated using two-sided t-tests in mixed-effects meta-regression models followed by robust variance estimation (RVE), based on the entire dataset of SOC observations (n = 258). CI, confidence interval. Carbon input source (living plants input, detrital input including litter/amendments, no input); experiment duration (from1 to 5,840 days), earthworm density (ind.m^-2^); earthworms functional types (endogeic, anecic, epigeic, and their mixtures), earthworms origin (native or invasive species), study type( field vs. laboratory incubation), ecosystem type (farmland, grassland, forest), and MAT.

| Factor | Estimate | CI | P value |
| --- | --- | --- | --- |
| carbon input source | — | — | 0.01 |
| Functional type | — | — | 0.28 |
| Earthworms origin | — | — | < 0.001 |
| Ecosystem type | — | — | 0.19 |
| Incubation type | — | — | 0.47 |
| Experiment duration | 0.028 | （0.01，0.046） | 0.004 |
| Density | 0.007 | （-0.014, 0.029） | 0.499 |
| MAT | -0.001 | （-0.032, 0.029） | 0.943 |
